# Sequential Stub Matching for Asymptotically Uniform Generation of Directed Graphs with a Given Degree Sequence

**DOI:** 10.1007/s00026-024-00715-0

**Published:** 2024-08-07

**Authors:** Femke van Ieperen, Ivan Kryven

**Affiliations:** Mathematical Institute, Utrecht University, PO Box 80010, 3508 TA Utrecht, The Netherlands

**Keywords:** Random graphs, Directed graphs, Randomised approximation algorithms, 05C80, 05C20, 68W20, 68W25

## Abstract

We discuss sequential stub matching for directed graphs and show that this process can be used to sample simple digraphs with asymptotically equal probability. The process starts with an empty edge set and repeatedly adds edges to it with a certain state-dependent bias until the desired degree sequence is fulfilled, whilst avoiding placing a double edge or self-loop. We show that uniform sampling is achieved in the sparse regime when the maximum degree $d_\text {max}$ is asymptotically dominated by $m^{1/4}$, where *m* is the number of edges. The proof is based on deriving various combinatorial estimates related to the number of digraphs with a given degree sequence and controlling concentration of these estimates in large digraphs. This suggests that sequential stub matching can be viewed as a practical algorithm for almost uniform sampling of digraphs. We show that this algorithm can be implemented to feature a linear expected runtime *O*(*m*).

## Introduction

In its simplest form, the problem of uniform graph sampling can be posed for undirected graphs. An undirected graph is considered simple when it has no multiple edges or self-loops. Given a degree sequence, the existence of the corresponding simple graph can be checked in linear time using the Erdős-Gallai criterion [15]; however, sampling simple graphs uniformly at random is difficult. One straightforward but computationally expensive way is rejection sampling with the configuration model [7]. The idea behind this method is to repeatedly perform random stub matching based on a given graphical degree sequence, whilst rejecting every final multigraph until eventually a simple graph is obtained. This procedure succeeds in time that is exponential in the square of the average degree [4]. The strategy can be improved if instead of rejecting every multigraph, one repairs them by switching edges to remove parallel edges. Such a procedure was shown to implement exact uniform sampling in linear time for undirected graphs in special regimes, for example, see [1].

### Beyond Undirected Graphs

Uniform generation of simple graphs is used in the analysis of algorithms and networks [26, 33, 34]. In algorithmic spectral graph theory, fast sampling is required to study spectra of sparse random matrices [25, 36, 38], where beyond the case of undirected graphs, heuristic algorithms reaching the output distribution had to be postulated. Moreover, generating random graphs is closely related to counting and generation of binary matrices with given row and column sums [9, 10], which can be interpreted as an adjacency or incidence matrix of a corresponding simple graph. In all of these areas, generation of *directed* graphs is of equal importance. Indeed, the adjacency matrix in the directed graph is non-symmetric, and hence it satisfies different column and row sums. Another reason to study directed random graphs is that, as a special case, they give a simple representation for bipartite graphs and hypergraphs that can be exploited for sampling [14]. To represent a simple bipartite graph (or a hypergraph), consider a digraph with all vertices being either sinks (identified with hypervertices) or sources (identified with hyperedges). Note that simple bipartite graphs are special cases of simple directed graphs but not the other way around: biadjacency matrix of a simple bipartite graph is an adjacency of a directed graph that is not necessarily simple. Nevertheless, one can use this correspondence for uniform sampling by additionally excluding perfect matchings.

### Related Work

Sampling of directed graphs, or graphs with certain constraints on their structure, has also been studied. Here we distinguish two of the main algorithmic families, whilst, for an exhaustive review, the reader is referred to Ref. [23].

Markov Chain Monte Carlo (MCMC) algorithms approximate the desired sample with a sequence (also called a chain) of approximate samples that improve the output distribution error with each iteration [5, 16, 21, 37, 41]. For this reason, initiating the MCMC sequence with a seed element that itself is chosen with the smallest error benefits such algorithms. At the same time, estimating how fast such chains converge to uniformity, the mixing time of the chain, is generally a difficult problem and has been achieved only for several classes of random graphs. Various MCMC algorithms were suggested for graphs with arbitrary degree sequences [17, 18, 28, 40], whereas the rapid mixing property was shown in Ref. [16] for the class of P-stable degree distributions, and more recently, for other stability classes by Gao and Greenhill [21]. For an example of convergence to uniformity analysis, see, e.g. Janson [27]. Bergerand and Müller-Hannemann suggested a MCMC algorithm for sampling random digraphs [5], with some relevant rapid mixing results shown by Greenhill [22, 24] and Erdős et al. [19]. Further generalisations were also proposed for degree-correlated random graphs and bipartite graphs [11, 14].

As an alternative to MCMC, sequential algorithms construct simple graphs by starting with an empty edge set and adding edges one-by-one with a stub matching. The crux is to employ the state-dependent importance sampling and select a new edge non-uniformly from the set of possible pairs whilst updating the probability after each edge placement [3, 29, 39]. Because the number of steps is fixed, sequential algorithms run in almost linear time. The price to pay is that the uniformity is achieved only asymptotically for large graphs. In practice, such methods are especially useful for assessing asymptotic properties of large graphs, e.g. to study sparse random matrices and complex networks. In principle, one should be able to eliminate the uniformity error even for a fixed number of vertices by first generating the seed graph with a sequential algorithm and then initiating an MCMC of choice with this seed. The other advantage is that sequential algorithms produce an a posteriori estimate for the total number of graphs with given constraints (e.g. degree sequence, bipartite structure), which makes them potentially useful for statistical inference [43].

Sequential sampling has been realised for regular graphs with the running time shown to be ${\mathcal {O}}(md_\text {max}^2)$ by Kim and Vu [29]. Bayati et al. [3] generalised the sequential method to an arbitrary degree sequence, yet maintaining a near-linear in the number of edges algorithmic complexity. For these algorithms, the maximum degree may depend on *n* with some asymptotic constraints, and the bounds on the error in the output distribution asymptotically vanish as *m* tends to infinity. Other approaches [2, 6, 12] realise non-uniform sampling whilst also outputting probability of the generated sample a posteriori. Hence, they may be used to compute expectations over the probability space of random graphs.

### Our Contribution

In this work, we provide a sequential algorithm for asymptotically uniform sampling of directed graphs with a given degree sequence. The samples are simple graphs, in the sense that they have no self-loops or parallel edges with identical orientation. By definition, when a di-graphical degree sequence is fixed (see Fulkerson criteria [20]), uniformly sampled digraphs satisfying it must have equal probability. Being only asymptotically uniform, our algorithm controls two sources of error when the largest degree is of the order $O(m^{1/4-\tau })$ for any $\tau >0$: (1) the total variation between the actual output distribution and the target uniform distribution vanishes for large graphs (or equivalently, apart from a negligible set of exceptions, the algorithm generates all graphs with asymptotically equal probability) and (2) although the algorithm may need to be restarted because it fails to produce a sample, this does not happen with probability 1 for large graphs. Amongst the benefits of our approach is fast expected runtime of *O*(*m*) as well as a priori (and a posteriori) estimates for the number of directed graphs with a given degree sequence. Note that a *priori* estimates for a similar setting are also available in Ref. [9].

We expect that introducing the transition from univariate degree distributions to degree distribution with two types of stubs (in- and out-edges) may open an avenue for further generalisations. For example, to sampling graphs with coloured edges or random geometric graphs [31, 32]. From the sampling perspective, coloured graphs comprise a less-tractable class of problems. For instance, even answering the question whether a given coloured degree sequence is graphical is an NP hard problem for more than two colours [8, 13]; nevertheless, it is reasonable to expect that randomised algorithms may perform such tests with high probability of success for degree sequences of sufficient regularity.

The algorithm is explained in Sect. 2. The proof that for a fixed degree sequence, the probability that the algorithm generates each graph, apart from a negligible set of exceptions, is a factor of $1 \pm o(1)$ times the uniform probability is presented in Sect. 3 and is inspired by the proof of Bayati, Kim and Saberi [3]. As in Ref. [3], the idea is showing a concentration of a certain random variable defined on the space of all orderings of *m* edges. The orderings encode the possible execution path of the sequential algorithm generating a given graph. To this end, we partition the space of all orderings into a system of cover sets, on which we separately study the concentration. To identify the value on which the random variable concentrates, we build a simpler random graph model in which the output graph is not dependent on the history of construction, and nevertheless show that the expected value of some key property in this model is also the value on which our random variable concentrates. Having two-component degree sequence, which defines in- and out-degrees for each vertex, requires more elaborate analysis when applying Vu’s concentration inequality [42]. Our algorithm may fail to construct a graph, but it is shown that this happens with probability *o*(1) in Sect. 4. The expected runtime analysis of the algorithm is shown to be *O*(*m*) in Sect. 5, which is faster than the estimate in Ref. [3] due to making use of the constant-time lookup in lists.

## Sequential Stub Matching

Our process for generating simple digraphs is best explained as a modification of the directed configuration model. This model generates a configuration by sequentially matching a random in-stub to a random out-stub. One can, therefore, see that generating a uniformly random configuration is not difficult; however, a random configuration may induce a multigraph, which is not desired. This issue can be remedied by the following procedure: At a given step, a single match between the chosen in- and out-stub is rejected if it leads to a self-loop or multi-edge. Then the resulting configuration necessarily induces a simple graph. Note that this rejection of specific matches destroys the uniformity of the generated graphs. To cancel out the non-uniformity bias, we accept each admissible match between an in- and an out-stub with a cleverly chosen probability, which restores asymptotically uniformity of the samples. Namely, we show that the distribution of the resulting graphs is within $1 \pm o(1)$ of uniformity for large graphs. Another consequence of the constraint on acceptable matches is that it may result in a failed attempt to finish a configuration; for example, if at some step of the matching procedure, the only remaining stubs consist of one in-stub and one out-stub belonging to the same vertex. In this case, we reject the entire configuration and start again from scratch. As we will show later in Sect. 5, a failure is not likely to occur, i.e. the probability that a configuration cannot be finished is *o*(1).

**Algorithm 1:**
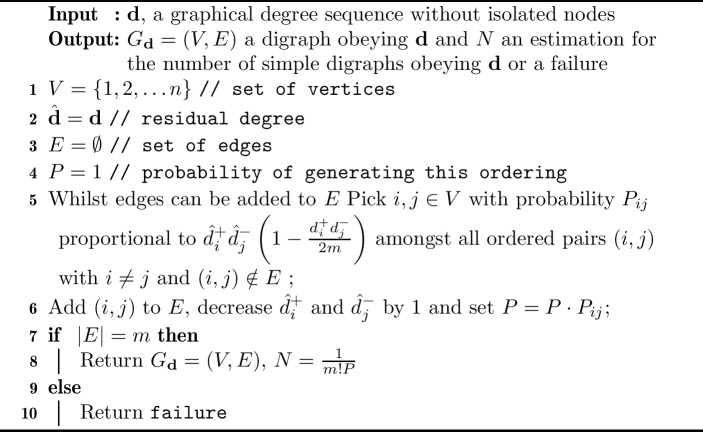
Generating simple directed graphs obeying a given degree sequence

The stub matching process can be formalised as pseudo-code, see Algorithm 1. We use the following notation: Let $\textbf{d}=\{({d^-_{ i}},{d^+_{i}})\}_{i=1}^n$ with ${d^-_{ i}},{d^+_{i}}\in {\mathbb {N}}$ be a di-graphical degree sequence with no isolated vertices, and $m=\sum _{i>0}{d^-_{ i}}=\sum _{i>0}{d^+_{i}}$ the total number of edges. Furthermore, we define

$$\begin{aligned} d_{\max } = \max \{\max \{{d^-_{ 1}},{d^-_{ 2}},\ldots ,{d^-_{ n}}\}, \max \{{d^+_{1}}, {d^+_{2}}, \ldots , {d^+_{n}}\} \}. \end{aligned}$$

We wish to construct a simple directed graph $G_{\textbf{d}}= \left( V,E\right)$ with vertex set $V=\{1,\dots ,n\}$ and edge set *E* whose degree sequence is $\textbf{d}$. At each step, Algorithm 1 chooses edge (*i*, *j*) with probability

$$\begin{aligned} P_{ij} \sim {\left\{ \begin{array}{ll} {\hat{d}}_i^+{\hat{d}}_j^-\left( 1-\frac{{d^+_{i}}{d^-_{ j}}}{2m}\right) ,&{} i\ne j \,\text {and}\, (i,j) \notin E,\\ 0,&{}i=j \,\text {or}\, (i,j) \in E, \end{array}\right. } \end{aligned}$$

and adds it to *E*, where the residual in-degree ${\hat{d}}_i^-$ (respectively residual out-degree ${\hat{d}}_i^+$) of vertex *i* is the number of unmatched in-stubs (out-stubs) of this vertex and *E* the set of edges constructed so far. If for all pairs $i,j \in V$ with ${\hat{d}}_i^+ >0$ and ${\hat{d}}_j^->0$ it happens that either $i=j$ or $(i,j) \in E$, no edge can be added to $E$ and the algorithm terminates. If the algorithm terminates before *m* edges have been added to *E*, it has failed to construct a simple graph obeying the desired degree sequence and outputs a *failure*. If the algorithm terminates with $|E|=m$, it returns a simple graph that obeys the degree sequence $\textbf{d}$ by construction. In this case, the algorithm also computes the total probability *P* of constructing the instance of $G_{\textbf{d}}$ in the given order of edge placement. We will show that almost all orderings of a set of *m* edges are generated with asymptotically equal probability. Hence, for almost all digraphs $G_{\textbf{d}}$, the probability that the algorithm generates them is asymptotically *m*!*P*. We will also show that digraphs are generated within a factor of $1+o(1)$ of uniformity, and therefore $N=\frac{1}{m!P}$ approximates the number of simple digraphs obeying the degree sequence. The algorithm returns the value of *N*, providing it has successfully terminated. To make these statements more precise, let us consider *degree progression* $\{\textbf{d}_n\}_{n\in {\mathbb {N}}}$, that is a sequence of graphical degree sequences indexed by the number of vertices *n*. The algorithm has the following favourable properties. Let the maximum degrees in $\{\textbf{d}_n\}_{n\in {\mathbb {N}}}$ satisfy $d_{\max ,n} = \mathcal {O}\left( m_n^{1/4 - \tau }\right)$ for an arbitrary $\tau >0$ and $m_n$ being the number of edges in $\textbf{d}_n$.

### Theorem 2.1

When applied to $\textbf{d}_n$, Algorithm 1 terminates successfully with probability $1 + o(1)$ and has the expected runtime of $\mathcal {O}\left( m\right)$.

### Theorem 2.2

Any $G_{\textbf{d}}$ is generated by Algorithm 1 with probability

$$\begin{aligned}&\mathbb {P}\left[ G_{\textbf{d}}\right] =\left( 1+o(1)\right) \frac{ \prod _{i=1}^n{d^+_{i}}! \prod _{i=1}^n {d^-_{ i}}!}{m!} \\&\quad e^{\frac{\sum _{i=1}^n {d^-_{ i}}{d^+_{i}}}{m} - \frac{\sum _{i=1}^n \left( ({d^-_{ i}})^2 + ({d^+_{i}})^2\right) }{2m}+ \frac{\sum _{i=1}^n({d^-_{ i}})^2\sum _{i=1}^n({d^+_{i}})^2}{2m^2} +\frac{1}{2}}. \end{aligned}$$

Note that the probability in Theorem 2.2 depends on the degree sequence but is asymptotically independent of $G_{\textbf{d}}$ itself, which indicates that almost all graphs that satisfy $\textbf{d}$ are generated with asymptotically equal probability. When considered outside of the context of Algorithm 1, this statement is similar to the recent result on asymptotic enumeration of digraphs [35], which can also be used to construct an alternative proof of the theorem. The remainder of this paper covers the proofs of Theorems 2.1 and 2.2, which are split into three parts, discussing the uniformity of the generated digraphs, the failure probability of the algorithm and its runtime.

## The Probability of Generating a Given Digraph

The goal of this section is to determine the probability $\mathbb {P}(G_{\textbf{d}})$ that Algorithm 1 outputs a given digraph $G_{\textbf{d}}$ on input of a graphical $\textbf{d}$, which will prove Theorem 2.2.

### Definition 3.1

Let $\textbf{d}$ be a degree sequence. For all $i \in \{1,2,\ldots , n\}$ define a set of *in-stubs* ${W^{-}_{i}}$ consisting of ${d^-_{ i}}$ unique elements and a set *out-stub* ${W^{+}_{i}}$ containing ${d^+_{i}}$ elements. Let ${W^{-}_{}}= \cup _{i \in \{1,2,\ldots , n\}} {W^{-}_{i}}$ and ${W^{+}_{}}= \cup _{ i \in \{1,2,\ldots , n\}}{W^{+}_{i}}$. Then a *configuration* is a perfect bipartite matching of ${W^{-}_{}}$ and ${W^{+}_{}}$, that is a set of tuples (*a*, *b*) such that each tuple contains one element from ${W^{-}_{}}$ and one from ${W^{+}_{}}$, and each element of ${W^{-}_{}}$ and ${W^{+}_{}}$ appears in exactly one tuple.

A configuration $\mathcal {M}$ prescribes a matching for all stubs and, therefore, defines a multigraph with vertices $V = \{1,2,\ldots , n\}$ and edge set

1 $$\begin{aligned} E= [ (i,j) \mid {W^{+}_{i}} \ni a, {W^{-}_{j}} \ni b, \text { and }(a,b) \in \mathcal {M}]. \end{aligned}$$

A fixed output of Algorithm 1 can be viewed as a configuration. Since the algorithm matches subs, there may be multiple stub pairs that lead to the same edge. Consequently, multiple configurations may lead to the same graph $G_{\textbf{d}}$.

Let $R(G_{\textbf{d}}) = \left\{ \mathcal {M}\,|\, G_\mathcal {M} = G_{\textbf{d}}\right\}$ be the set of all configurations on $\left( {W^{-}_{}}, {W^{+}_{}}\right)$ that correspond to $G_{\textbf{d}}$. The probability that the algorithm generates $G_{\textbf{d}}$ is the sum of probabilities that the algorithm generates a configuration that correspond to $G_{\textbf{d}}$,

$$\begin{aligned} \mathbb {P}(G_{\textbf{d}}) = \sum _ { \mathcal {M} \in R(G_{\textbf{d}})} \mathbb {P}\left( \mathcal {M}\right) . \end{aligned}$$

Different configurations correspond to the same graph if they differ only in the labelling of the stubs. Since the probability to match an out-stub of *i* to an in-stub of *j* at a given step of the algorithm depends on the partial configuration constructed so far, the order in which the matches are chosen influences the probability of generating a configuration $\mathcal {M}$. Let for a given $\mathcal {M} \in R(G_{\textbf{d}})$, $S\left( \mathcal {M}\right)$ be the set of all the orderings $\mathcal {N}$ in which this configuration can be created. Because the configuration already determines the match for each in-stub, an ordering of $\mathcal {M}$ can be thought of as an enumeration of edges $\mathcal {N} = \left( e_1,e_2,\ldots , e_m\right) ,\;e_i\in E,$ defining which in-stub gets matched first, which second, etc. There are *m*! different orderings of the configuration $\mathcal {M}$. This implies that for any $\mathcal {M} \in R(G_{\textbf{d}})$,

$$\begin{aligned} \mathbb {P}(G_{\textbf{d}}) = \prod _{i=1}^{n}d_i^{-}! \prod _{i=1}^{n}d_i^{+}!\sum _{ \mathcal {N} \in S\left( \mathcal {M}\right) } \mathbb {P}\left( \mathcal {N}\right) . \end{aligned}$$

Hence, we further investigate $\mathbb {P}\left( \mathcal {N}\right)$. If the algorithm has constructed the first *r* elements of $\mathcal {N}$, it is said to be at step $r \in \{0,1,\ldots , m-1\}$. There is no step *m*, as the algorithm terminates immediately after constructing the $m^\text {th}$ edge. Let ${{d^-_{ i}}}^{(r)}$ (respectively ${{d^+_{i}}}^{(r)}$) denote the number of unmatched in-stubs (out-stubs) of the vertex *i* at step *r*. Let $E_r$ be the set of admissible edges that can be added to the ordering at step *r*,

2 $$\begin{aligned} E_r:= \left\{ (i,j) \mid i,j \in V, \; {{d^+_{i}}}^{(r)}>0, \; {{d^-_{ j}}}^{(r)}>0, \; i\ne j, \; (i,j) \notin \{e_1, e_2 \ldots , e_r\}\right\} . \nonumber \\ \end{aligned}$$

With this notation in mind, we write the probability of generating the entire ordering $\mathcal {N}$ as

$$\begin{aligned} \mathbb {P}(\mathcal {N}) = \prod _{r=0}^{m-1} \mathbb {P}\left[ e_{r+1}= (i,j) | e_1, \ldots , e_r\right] , \end{aligned}$$

where

$$\begin{aligned} \mathbb {P}\left[ e_{r+1} = (i,j) | e_1, \ldots , e_r\right] = \frac{1-\frac{{d^+_{i}}{d^-_{ j}}}{2m}}{\sum _{(u,v) \in E_r} {{d^+_{u}}}^{(r)}{{d^-_{ v}}}^{(r)}\left( 1 - \frac{{d^+_{u}}d_v^-}{2m}\right) }. \end{aligned}$$

is the conditional probability that a given out-stub of *i* is matched with a given in-stub of *j*. Noticing that $\sum _{u\in V}{{d^+_{u}}}^{(r)}=\sum _{u\in V}{{d^-_{ u}}}^{(r)}=m-r$, the probability that the algorithm generates the graph $G_{\textbf{d}}$ can be written as

3 $$\begin{aligned} \mathbb {P}(G_{\textbf{d}}) = \prod _{i=1}^n {d^-_{ i}}! \prod _{i=1}^n{d^+_{i}}! \prod _{(i,j) \in G_{\textbf{d}}} \left( 1 - \frac{{d^+_{i}}{d^-_{ j}}}{2m} \right) \sum _ {\mathcal {N} \in S(\mathcal {M})} \prod _{r=0}^{m-1} \frac{1}{(m-r)^2 - \Psi _r(\mathcal {N})}, \end{aligned}$$

where

4 $$\begin{aligned} \Psi _r\left( \mathcal {N}\right) = \sum _{(u,v) \notin E_r}{{d^+_{u}}}^{(r)}{{d^-_{ v}}}^{(r)} + \sum _{(u,v) \in E_r} {{{d^+_{u}}}^{(r)}} {{{d^-_{ v}}}^{(r)}} \frac{{d^+_{u}}d_v^-}{2m}. \end{aligned}$$

where $(u,v) \notin E_r$ is a set of stub pairs at step *r* that are unsuitable because they correspond to a self-loop or parallel edge. By formally comparing the expression (3) with the statement of Theorem 2.2, we observe that to complete the proof, it is sufficient to show that for any $G_{\textbf{d}}$, $\Psi _r\left( \mathcal {N}\right)$ sharply concentrates on some $\psi _r$ dependent on $\textbf{d}$ but not the the structure $G_{\textbf{d}}$ itself. Namely,

5 $$\begin{aligned} \sum _{ \mathcal {N} \in S\left( \mathcal {M}\right) } \prod _{r=0}^{m-1} \frac{1}{(m-r)^2 - \Psi _r(\mathcal {N})} = \left[ 1 + o(1)\right] m! \prod _{r=0}^{m-1} \frac{1}{(m-r)^2 - \psi _r}, \end{aligned}$$

and

6 $$\begin{aligned} \begin{aligned}&\prod _{r=0}^{m-1} \frac{1}{(m-r)^2 - \psi _r} =\left[ 1+o(1)\right] \left( \prod _{r=0}^{m-1} \frac{1}{(m-r)^2} \right) \\&\quad e^{\frac{\sum _{i=1}^n {d^-_{ i}}{d^+_{i}}}{m} - \frac{\sum _{i=1}^n ({d^-_{ i}})^2 + ({d^+_{i}})^2}{2m} +\frac{\sum _{i=1}^n({d^-_{ i}})^2\sum _{i=1}^n({d^+_{i}})^2}{2m^2} + \frac{\sum _{(i,j) \in G_{\textbf{d}}} {d^+_{i}}{d^-_{ j}}}{2m} + \frac{1}{2}}. \end{aligned} \nonumber \\ \end{aligned}$$

Indeed, combining the latter two equations with (3) and using that $1-x = e^{-x + \mathcal {O}(x^2)}$ and $\prod _{r=0}^{m-1} (m-r)^2=(m!)^2$ we find:

$$\begin{aligned}&\mathbb {P}(G_{\textbf{d}}) = \left[ 1+o(1)\right] \frac{\prod _{i=1}^n {d^-_{ i}}! \prod _{i=1}^n{d^+_{i}}! }{m!}\\&\quad e^{\frac{\sum _{i=1}^n {d^-_{ i}}{d^+_{i}}}{m} - \frac{\sum _{i=1}^n ({d^-_{ i}})^2 + ({d^+_{i}})^2}{2m} + \frac{\sum _{i=1}^n({d^-_{ i}})^2\sum _{i=1}^n({d^+_{i}})^2}{2m^2} + \frac{1}{2}}, \end{aligned}$$

which coincides with the statement of Theorem 2.2. Thus, proving Eqs. (5) and (6) suffices to show validity of Theorem 2.2.

### Defining $\psi _r$

Fix $G_{\textbf{d}}$ with *m* edges and let $p_r: = \frac{r}{m}$, $r\in [m]$. Consider a random graph $G_{p_r}$ in which each edge of $G_{\textbf{d}}$ is independently present with identical probability $p_r$. In other words, $G_{p_r}$ is the edge percolation of some fixed $G_{\textbf{d}}$. For given $p_r$, a realisation of this random graph $\mathcal {G}\sim G_{p_r}$ can be compared to the partially constructed graph at step *r* by Algorithm 1. In the similar fashion, applying definition (2) directly to $\mathcal {G}$ as subgraph of $G_{\textbf{d}}$ gives the set of admissible edges generated by random model $G_{p_r}$. We refer to this set as $E_{p_r}$. The idea now is that at a given step *r*, we can compute the corresponding $p_r$ and, hence, study the expectation of some function of $E_{p_r}$ and compare this expectation to the expectation of the actual function on $E_r$. For example, we can compare the expected number of unsuitable pairs as generated by the algorithm and the random model. To distinguish the two probability spaces, we use subindices *r* versus $p_r$. The final goal of this section is to compare $\Psi _r$, as defined in Eq. (4), to $\psi _r: = \mathbb {E}_{p_r}\left[ \Psi _r\right]$ —the expected value of the quantity from definition (4) computed in model $G_{p_r}$.

Recall that ordering $\mathcal {N}$ is chosen uniformly at random from all orderings of configuration *M*. Note further that, as an output of our randomised algorithm, $\Psi _r\left( \mathcal {N}\right)$ can be viewed as a function on the subgraph of $G_{\textbf{d}}$ induced by the first *r* elements of the ordering $\mathcal {N}$, which we denote by $G_{\mathcal {N}_r}$. The goal is to show that random variable $\Psi _r\left( \mathcal {N}\right)$, which is otherwise hard to study because of its dependency on the pervious steps, concentrates on $\psi _r$, which is a simpler object. The only parameter connecting the two models is $p_r.$ We abbreviate $\Psi _r$ as $\Psi _r(\mathcal {N})$ whenever $\mathcal {N}$ follows from the context.

Let us split $\Psi _r$, as defined in Eq. (4), into a sum of two terms:

$$\begin{aligned} \Psi _r = \Delta _r + \Lambda _r, \end{aligned}$$

with

7 $$\begin{aligned} \Delta _r = \sum _{(u,v) \notin E_r}{{d^+_{u}}}^{(r)}{{d^-_{ v}}}^{(r)} \quad \text {and} \quad \Lambda _r = \sum _{(u,v) \in E_r} {{{d^+_{u}}}^{(r)}} {{{d^-_{ v}}}^{(r)}} \frac{{d^+_{u}}d_v^-}{2m}. \end{aligned}$$

Here $\Delta _r$ counts the number of *unsuitable pairs* at step *r*, i.e. the number of pairs of the unmatched in-stubs with out-stubs that will induce a self-loop or multi-edge if added, and $\Lambda _r$ counts the number of suitable pairs (multiplied by the importance sampling factor). In the sequel, we refer to a combination of an unmatched in- and out-stub as a *pair*. Furthermore, we also split

$$\begin{aligned} \Delta _r = \Delta _r^1 + \Delta _r^2, \end{aligned}$$

into the sum of the number pairs leading to self-loops, $\Delta _r^1 = \sum _{i=1}^n {{d^-_{ i}}}^{(r)}{{d^+_{i}}}^{(r)}$, and the number of pairs leading to double edges, $\Delta _r^2 = \Delta _r - \Delta _r^1.$ For the suitable pairs, we split

$$\begin{aligned} \Lambda _r = \frac{{\Lambda _r^1}^+{\Lambda _r^1}^- - \Lambda _r^2}{2m} - \frac{\Lambda _r^3}{2m}, \end{aligned}$$

where

8 $$\begin{aligned}&{\Lambda _r^1}^+ = \sum _{i=1}^n {{{d^+_{i}}}^{(r)}}{d^+_{i}}, \quad {\Lambda _r^1}^- = \sum _{i=1}^n {{d^-_{ i}}}^{(r)}{d^-_{ i}}, \end{aligned}$$

9 $$\begin{aligned}&\Lambda _r^2 = \sum _{i=1}^n {{{d^+_{i}}}^{(r)}}{d^+_{i}}{{d^-_{ i}}}^{(r)}{d^-_{ i}}, \end{aligned}$$

10 $$\begin{aligned}&\Lambda _r^3 = \sum _{\mathop {\begin{array}{c} (u,v)\notin E_r\\ u\ne v \end{array}}} {{{d^+_{u}}}^{(r)}}{{{d^-_{ v}}}^{(r)}}{d^+_{u}}d_v^-. \end{aligned}$$

Here $\frac{{\Lambda _r^1}^+{\Lambda _r^1}^-}{2m}$ relates to total number of possible pairs in the whole graph, $\frac{{\Lambda _r^1}^+{\Lambda _r^1}^- - \Lambda _r^2}{2m}$ subtracts pairs that are self-loops, $\frac{\Lambda _r^3}{2m}$ further reduces this quality by already matched edges to obtain suitable pairs. We will now derive several bounds on the latter quantities, to be used in Sect. 3.3.

#### Lemma 3.2

For all $$0 \le r \le m-1$$,

(i) $$\Delta _r \le (m-r)d_{\max }^2$$;
(ii) $${\Lambda _r^1}^+ \le d_{\max }(m-r), \;{\Lambda _r^1}^- \le d_{\max }(m-r)$$;
(iii) $$\Lambda _r \le \frac{d_{\max }^2}{2m}(m-r)^2$$.

#### Proof

(i) At step *r*, there are $m-r$ unmatched in-stubs left. Each unmatched in-stub can form a self-loop by connecting to an unmatched out-stub of the same vertex. The number of unmatched out-stubs at each vertex is upper bounded by $d_{\max }$, hence $\Delta _r^1 \le (m-r)d_{\max }$. The vertex to which an unmatched in-stub belongs has at most $d_{\max }-1$ incoming edges. The source of such an edge has at most $d_{\max }-1$ unmatched out-stubs left. Thus, the number of out-stubs an unmatched in-stub can be paired with to create a double edge is at most $\left( d_{\max }-1\right) ^2$. Hence $\Delta _r^2 \le (m-r)(d_{\max } - 1)^2$ and $\Delta _r = \Delta _r^1 + \Delta _r^2 \le (m-r)d_{\max }^2$.
(ii) By definition, ${\Lambda _r^1}^+ = \sum _{i=1}^n {{d^+_{i}}}^{(r)}{d^+_{i}}$. As $\sum _{i=1}^n {{d^+_{i}}}^{(r)} = m-r$ and ${d^+_{i}} \le d_{\max }$ for all *i*, this implies that ${\Lambda _r^1}^+ \le d_{\max }(m-r)$ and ${\Lambda _r^1}^- \le d_{\max }(m-r)$.
(iii) By definition, $\Lambda _r = \sum _{(u,v) \in E_r} {{d^+_{u}}}^{(r)}{{d^-_{ v}}}^{(r)}\frac{{d^+_{u}}d_v^-}{2m} \le \frac{d_{\max }^2}{2m}\sum _{(u,v) \in E_r} {{d^+_{u}}}^{(r)}{{d^-_{ v}}}^{(r)}$. Since $\sum _{i=1}^n {{d^+_{u}}}^{(r)} = m-r$ and ${{d^-_{ v}}}^{(r)} \le (m-r)$ for all *v*, the claim follows.

$$\square $$

Let us consider some basic properties of random variables ${{d^+_{i}}}^{(r)}$ and ${{d^-_{ i}}}^{(r)}$ in model $G_{p_r}$. Recall that by definition, the value of ${{d^+_{i}}}^{(r)}$ equals the number of edges $(i, \bullet ) \in G_{\textbf{d}}$, such that $(i, \bullet ) \notin G_{p_r}$. Since $G_{\textbf{d}}$ is simple and has no self-loops, ${{d^-_{ i}}}^{(r)}$ and ${{d^+_{i}}}^{(r)}$ are independent. Moreover, ${{d^+_{i}}}^{(r)}$ and ${{d^-_{ j}}}^{(r)}$ are independent, unless $(i,j) \in G_{\textbf{d}}$. Indeed, ${{d^+_{i}}}^{(r)}$ (respectively ${{d^-_{ j}}}^{(r)}$) is the sum of ${d^+_{i}}$ (${d^-_{ j}}$) independent Bernoulli variables representing the out-stubs (in-stubs). Furthermore, we will use subindex $p_r$ to refer to a random variable in $G_{p_r}$ that corresponds to one of the deterministic quantities defined above. For example, $\Delta _{p_r}$ is a random variable corresponding to $\Delta _{r}$, with $p_r=\frac{r}{m}$. Next, the following expected values are defined with respect to random graph model $G_{p_r}$, again the parameter *r* should be understood as a quantity in $G_{p_r}$ that has the same partially completed degree sequence as the corresponding quantity in $G_{\mathcal {N}_r}$:

#### Lemma 3.3

For each $0 \le r \le m-1$, the following equations hold:

(i) $$\mathbb {E}_{p_r}\!\left[ \Delta _r^1\right] = \frac{(m-r)^2}{m^2}\sum _{i=1}^n{d^+_{i}}{d^-_{ i}}$$;
(ii) $$\mathbb {E}_{p_r}\!\left[ \Delta _r^2\right] = \frac{r(m-r)^2}{m^3} \sum _{(i,j) \in G_{\textbf{d}}}({d^+_{i}}-1)({d^-_{ j}}-1)$$;
(iii) $$\mathbb {E}_{p_r}\!\left[ {\Lambda _r^1}^-{\Lambda _r^1}^+\right] = \frac{(m-r)^2}{m^2} \sum _{i=1}^n ({d^-_{ i}})^2 \sum _{i=1}^n({d^+_{i}})^2 + \frac{r(m-r)}{m^2} \sum _{(i,j) \in G_{\textbf{d}}} {d^+_{i}}{d^-_{ j}}$$;
(iv) $$\mathbb {E}_{p_r}\!\left[ \Lambda _r^2\right] = \frac{(m-r)^2}{m^2}\sum _{i=1}^n ({d^-_{ i}})^2({d^+_{i}})^2$$;
(v) $$\mathbb {E}_{p_r}\!\left[ \Lambda _r^3\right] = \frac{r(m-r)^2}{m^3} \sum _{(i,j) \in G_{\textbf{d}}}{d^+_{i}}({d^+_{i}}-1){d^-_{ j}}({d^-_{ j}}-1)$$.

#### Proof

(i) Since $p_r = \frac{r}{m}$, we have $\mathbb {E}_{p_r}\!\left[ {d_i^{\pm }}^{(r)}\right] = d_i^{\pm }\frac{m-r}{m}$. Using the facts that ${{d^-_{ i}}}^{(r)}$ and ${{d^+_{i}}}^{(r)}$ are independent and $\Delta _r^1 = \sum _{i=1}^n {{d^-_{ i}}}^{(r)}{{d^+_{i}}}^{(r)}$, we find $\mathbb {E}_{p_r}\!\left[ \Delta _r^1\right] = \frac{(m-r)^2}{m^2}\sum _{i=1}^n{d^+_{i}}{d^-_{ i}}$.
(ii) $\Delta _r^2$ counts the number of pairs, such that if they were chosen at step *r*, we would receive a double edge. Fix step *r* and a realisation of $G_{p_r}$, and choose a pair $(i,j) \in G_{\textbf{d}}$. Edge (*i*, *j*) leads to a double edge at the current step of the algorithm if (*i*, *j*) is already present in $G_{p_r}$. This can only happen if there are edges (*i*, *k*) and (*l*, *j*) that are in $G_{\textbf{d}}$ but not in $G_{p_r}$. This means that in $G_{p_r}$, there are unmatched in-stubs and out-stubs such that if one were to match the edges (*i*, *j*) and (*l*, *k*), one obtains a double edge. The number of combinations of such *l* and *k*, is $({{d^+_{i}}}^{(r)}-1)({{d^-_{ j}}}^{(r)}-1)$. Because of independence of ${{d^+_{i}}}^{(r)}$ and ${{d^-_{ i}}}^{(r)}$, the expectation of $\Delta _r^2$ is a sum of the latter quantity over all edges of $G_{\textbf{d}}$ multiplied by probability $p_r$.
(iii) Remark that ${\Lambda _r^1}^-{\Lambda _r^1}^+ = \sum _{j=1}^n\sum _{i=1}^n {{d^+_{i}}}^{(r)}{{d^-_{ j}}}^{(r)}{d^+_{i}}{d^-_{ j}}$, which implies that
  $$\begin{aligned} \mathbb {E}_{p_r}\!\left[ {\Lambda _r^1}^-{\Lambda _r^1}^+\right] = \sum _{j=1}^n\sum _{i=1}^n \mathbb {E}_{p_r}\!\left[ {{d^+_{i}}}^{(r)}{{d^-_{ j}}}^{(r)}\right] {d^+_{i}}{d^-_{ j}}. \end{aligned}$$
  Recall that ${{d^+_{i}}}^{(r)}$ (respectively ${{d^-_{ j}}}^{(r)}$) is the sum of ${d^+_{i}}$ (${d^-_{ j}}$) independent Bernoulli variables representing the out-stubs (in-stubs). If $(i,j) \in G_{\textbf{d}}$, one fixed in-stub of *j* forms an edge with a fixed out-stub of *i*. This implies that the corresponding Bernoulli variables always need to take on the same value. Let us denote these Bernoulli variables by $d_{i_j}^+$ and $d_{j_i}^-$. Then, $\mathbb {E}_{p_r}\!\left[ {{d^+_{i}}}^{(r)}{{d^-_{ j}}}^{(r)} \right] = \mathbb {E}_{p_r}\!\left[ {{d^+_{i}}}^{(r)}\right] \mathbb {E}_{p_r}\!\left[ {{d^-_{ j}}}^{(r)}\right] + \text {Cov}\left( {{d^+_{i}}}^{(r)}{{d^-_{ j}}}^{(r)}\right)$. As already explained in (*i*) $\mathbb {E}_{p_r}\!\left[ {{d^+_{i}}}^{(r)}\right] \mathbb {E}\left[ {{d^-_{ j}}}^{(r)}\right] = \frac{(m-r)^2}{m^2}{d^+_{i}}{d^-_{ j}}$. For the covariance we have
  $$\begin{aligned} \text {Cov}\left( {{d^+_{i}}}^{(r)}{{d^-_{ j}}}^{(r)}\right) = {\left\{ \begin{array}{ll} 0 &{} \text {if}\, (i,j) \notin G_{\textbf{d}}\\ \text {Cov}\left( d_{i_j}^+{d}_{j_i}^-\right) &{} \text {if} \, (i,j) \in G_{\textbf{d}}\end{array}\right. }. \end{aligned}$$
  For the covariance of an arbitrary random variable *X* and a Bernoulli variable *Y* with expectation $p^*$, we have $\text {Cov}\left( X,Y\right) = \left( \mathbb {E}\left[ X|Y=1\right] \right. \left. - \mathbb {E}\left[ X|Y=0\right] \right) p^*(1-p^*)$. Applying this to $X= d_{i_j}^+$ and $Y=d_{j_i}^-$, their covariance becomes $\frac{r(m-r)}{m^2}$. Thus,
  $$\begin{aligned} \mathbb {E}_{p_r}\!\left[ {{d^+_{i}}}^{(r)}{{d^-_{ j}}}^{(r)}\right] = {\left\{ \begin{array}{ll} \frac{(m-r)^2}{m^2}{d^+_{i}}{d^-_{ j}} &{} \text {if} \, (i,j) \notin G_{\textbf{d}}\\ \frac{(m-r)^2}{m^2}{d^+_{i}}{d^-_{ j}} + \frac{r(m-r)}{m^2} &{} \text {if} \, (i,j) \in G_{\textbf{d}}\end{array}\right. }. \end{aligned}$$
  Plugging this back into the expression for $\mathbb {E}_{p_r}\!\left[ {\Lambda _r^1}^-{\Lambda _r^1}^+\right]$ the desired equation follows.
(iv) Recall that $\Lambda _r^2 = \sum _{i=1}^n {{d^-_{ i}}}^{(r)}{{d^+_{i}}}^{(r)}{d^-_{ i}}{d^+_{i}}$. In the proof of (*i*), we have already showed that $\mathbb {E}_{p_r}\!\left[ {{d^-_{ i}}}^{(r)}{{d^+_{i}}}^{(r)}\right] = {d^+_{i}}{d^-_{ i}} \frac{(m-r)^2}{m^2}$. Hence $\mathbb {E}_{p_r}\!\left[ \Lambda _r^2\right] = \frac{(m-r)^2}{m^2}\sum _{i=1}^n {d^-_{ i}}^2{d^+_{i}}^2$.
(v) From Eq. (10), it follows that $\Lambda _r^3 = \sum _{(i,j) \notin E_r, i \ne j} {d^+_{i}}{d^-_{ j}} {{d^+_{i}}}^{(r)}{{d^-_{ j}}}^{(r)}$.
  Since that $\Delta _r^2 = \sum _{(i,j) \notin E_r, i \ne j} {{d^+_{i}}}^{(r)}{{d^-_{ j}}}^{(r)}$, we can use the proof of (*ii*). This implies each edge $(i,j) \in G_{\textbf{d}}$ contributes $\frac{(m-r)^2}{m^2}\frac{r {d^+_{i}}({d^+_{i}}-1){d^-_{ j}}({d^-_{ j}}-1)}{m}$ to the sum, proving the claim.

$$\square $$

Next, we will use the following asymptotic estimates,

- $$ \sum _{i=1}^n \left( {d^-_{ i}}\right) ^s = \sum _{(i,j) \in G_{\textbf{d}}} \left( {d^-_{ i}}\right) ^{s-1} = \mathcal {O}\left( m d_{\max }^{s-1}\right) $$,
- $$ \sum _{i=1}^n \left( {d^+_{i}}\right) ^t = \sum _{(i,j) \in G_{\textbf{d}}} \left( {d^+_{i}}\right) ^{t-1} = \mathcal {O}\left( m d_{\max }^{t-1}\right) $$,
- $$ \sum _{i=1}^n \left( {d^-_{ i}}\right) ^s\left( {d^-_{ i}}\right) ^t = \sum _{(i,j) \in G_{\textbf{d}}} \left( {d^-_{ i}}\right) ^{s-1}\left( {d^+_{i}}\right) ^t = \mathcal {O}\left( m d_{\max }^{s+t-1}\right) $$,

to obtain and approximation of $\psi _r$ that we will work with. Combing these estimates with Lemma 3.3, we find

$$\begin{aligned}&\mathbb {E}_{p_r}\!\left[ \frac{{\Lambda _r^1}^-{\Lambda _r^1}^+}{2m}\right] = \frac{(m-r)^2}{2m^3} \sum _{i=1}^n ({d^-_{ i}})^2 \sum _{i=1}^n({d^+_{i}})^2 + (m-r)^2\mathcal {O}\left( \frac{rd_{\max }^2}{(m-r)m^2}\right) , \\&\mathbb {E}_{p_r}\!\left[ \frac{\Lambda _r^2}{2m}\right] = (m-r)^2 \mathcal {O}\left( \frac{d_{\max }^3}{m^2}\right) \quad \text {and}\quad \mathbb {E}_{p_r}\!\left[ \frac{\Lambda _r^3}{2m}\right] = (m-r)^2 \mathcal {O}\left( r\frac{d_{\max }^4}{m^3}\right) . \end{aligned}$$

This allows us to state the following Lemmas, which will be useful in Sects. 3.2 and 3.3.

#### Lemma 3.4

For all $$0 \le r \le m-1$$,

11 $$\begin{aligned} \psi _r&= (m-r)^2 \left[ \frac{\sum _{i=1}^n {d^-_{ i}}{d^+_{i}}}{m^2} + \frac{r\sum _{(i,j) \in G_{\textbf{d}}} \left( {d^+_{i}}-1\right) \left( {d^-_{ j}}-1\right) }{m^3} \right. \nonumber \\&\Bigg .\quad + \frac{\sum _{i=1}^n ({d^-_{ i}})^2 \sum _{i=1}^n({d^+_{i}})^2}{2m^3} + \xi _r\Bigg ], \end{aligned}$$

with error term $\xi _r = \mathcal {O}\left( \frac{d_{\max }^3}{m^2}+ \frac{rd_{\max }^2}{(m-r)m^2} + \frac{rd_{\max }^4}{m^3}\right)$.

#### Lemma 3.5

For each $0 \le r \le m-1$, the quantity $\psi _r$ is upper bounded by $\mathcal {O}\left( (m-r)^2 \frac{d_{\max }^2}{m}\right)$.

#### Proof

Combing Eq. (11) with the asymptotic estimate

$$\begin{aligned} \sum _{i=1}^n \left( {d^-_{ i}}\right) ^s\left( {d^-_{ i}}\right) ^t = \sum _{(i,j) \in G_{\textbf{d}}} \left( {d^-_{ i}}\right) ^{s-1}\left( {d^+_{i}}\right) ^t = \mathcal {O}\left( m d_{\max }^{s+t-1}\right) \end{aligned}$$

we find that $\psi _r = (m-r)^2\mathcal {O}\left( \frac{d_{\max }}{m} + \frac{rd_{\max }^2}{m^2} + \frac{d_{\max }^2}{2\,m} + \frac{rd_{\max }^2}{(m-r)m^2} + \frac{d_{\max }^3}{m^3}+\frac{rd_{\max }^4}{m^3}\right) ,$ and since $r \le m$ and $d_{\max }^2 = o(m),$ the latter equation becomes

$$\begin{aligned} \psi _r=(m-r)^2\mathcal {O}\left( \frac{d_{\max }^2}{m}\right) . \end{aligned}$$

$$\square $$

### Proof of Eq. (6)

With the help of Lemmas 3.4 and 3.5, we are now ready to prove Eq. (6). We start by multiplying the left hand side of Eq. (6) by $\prod _{r=0}^{m-1}(m-r)^2$:

$$\begin{aligned}&\prod _{r=0}^{m-1} \frac{(m-r)^2}{(m-r)^2 - \psi _r} = \prod _{r=0}^{m-1} \left( 1 + \frac{\psi _r}{(m-r)^2 - \psi _r}\right) . \end{aligned}$$

Applying Lemma 3.4 to the numerator and Lemma 3.5 to the denominator, the right hand side of the latter equation becomes:

$$\begin{aligned}&\exp \left[ \sum _{r=0}^{m-1} \ln \left( 1 + \frac{\frac{\sum _{i=1}^n {d^-_{ i}}{d^+_{i}}}{m^2} + \frac{r\sum _{(i,j) \in G_{\textbf{d}}} \left( {d^+_{i}}-1\right) \left( {d^-_{ j}}-1\right) }{m^3} + \frac{\sum _{i=1}^n ({d^-_{ i}})^2 \sum _{i=1}^n({d^+_{i}})^2}{2m^3} + \xi _r}{1 - \mathcal {O}\left( \frac{d_{\max }^2}{m}\right) }\right) \right] . \end{aligned}$$

Using $\frac{1}{1-x} = 1 + x + \mathcal {O}\left( x^2\right)$, $\ln (1+x) = x - \mathcal {O}(x^2)$ and $\mathcal {O}\left( \frac{d_{\max }^2}{m}\right) = \mathcal {O}\left( \frac{1}{m^{1/2 + 2\tau }}\right)$, we obtain the chain of estimates

$$\begin{aligned}&= \text {exp}\left[ \sum _{r=0}^{m-1} \ln \left( 1 + \frac{\sum _{i=1}^n {d^-_{ i}}{d^+_{i}}}{m^2} + \frac{r\sum _{(i,j) \in G_{\textbf{d}}} \left( {d^+_{i}}-1\right) \left( {d^-_{ j}}-1\right) }{m^3} \right. \right. \\&\Bigg .\left. \quad + \frac{\sum _{i=1}^n ({d^-_{ i}})^2 \sum _{i=1}^n({d^+_{i}})^2}{4m^3} + \xi _r \right) \Bigg ]\\&= \text {exp}\left[ \sum _{r=0}^{m-1} \frac{\sum _{i=1}^n {d^-_{ i}}{d^+_{i}}}{m^2} + \frac{r\sum _{(i,j) \in G_{\textbf{d}}} \left( {d^+_{i}}-1\right) \left( {d^-_{ j}}-1\right) }{m^3}\right. \\&\Bigg .\quad + \frac{\sum _{i=1}^n ({d^-_{ i}})^2 \sum _{i=1}^n({d^+_{i}})^2}{4m^3} + \mathcal {O}\left( \frac{d_{\max }^4}{m^2} + \frac{rd_{\max }^4}{m^3} + \frac{rd_{\max }^2}{(m-r)m^2}\right) \Bigg ] \\&= \text {exp}\left[ \frac{\sum _{i=1}^n {d^-_{ i}}{d^+_{i}}}{m} + (m-1)\frac{\sum _{(i,j) \in G_{\textbf{d}}} \left( {d^+_{i}}-1\right) \left( {d^-_{ j}}-1\right) }{2m^2} \right. \\&\Bigg .\quad + \frac{\sum _{i=1}^n ({d^-_{ i}})^2 \sum _{i=1}^n({d^+_{i}})^2}{4m^2} + \mathcal {O}\left( \frac{d_{\max }^4}{m} + \frac{d_{\max }^2}{m}\ln (m)\right) \Bigg ]\\&= \text {exp}\left[ \frac{\sum _{i=1}^n {d^-_{ i}}{d^+_{i}}}{m} + \frac{\sum _{(i,j) \in G_{\textbf{d}}} \left( {d^+_{i}}-1\right) \left( {d^-_{ j}}-1\right) }{2m}\right. \\&\quad \Bigg . + \frac{\sum _{i=1}^n ({d^-_{ i}})^2 \sum _{i=1}^n({d^+_{i}})^2}{4m^2} + o(1) \Bigg ] \\&= \text {exp}\left[ \frac{\sum _{i=1}^n {d^-_{ i}}{d^+_{i}}}{m} - \frac{\sum _{i=1}^n ({d^-_{ i}})^2 +\sum _{i=1}^n ({d^+_{i}})^2 }{2m} \right. \\&\Bigg .\quad + \frac{\sum _{i=1}^n ({d^-_{ i}})^2 \sum _{i=1}^n({d^+_{i}})^2}{4m^2} + \frac{\sum _{(i,j) \in G_{\textbf{d}}} {d^+_{i}}{d^-_{ j}}}{2m} + \frac{1}{2} + o(1) \Bigg ]. \end{aligned}$$

Thus, we have shown that

$$\begin{aligned} \begin{aligned} \prod _{r=0}^{m-1} \frac{(m-r)^2}{(m-r)^2 - \psi _r}&= \left[ 1+o(1)\right] \exp \Bigg [ \frac{\sum _{i=1}^n {d^-_{ i}}{d^+_{i}}}{m} - \frac{\sum _{i=1}^n ({d^-_{ i}})^2 +\sum _{i=1}^n ({d^+_{i}})^2 }{2m} \Bigg . \\&\quad \Bigg . +\frac{\sum _{i=1}^n ({d^-_{ i}})^2 \sum _{i=1}^n({d^+_{i}})^2}{2m^2} + \frac{\sum _{(i,j) \in G_{\textbf{d}}} {d^+_{i}}{d^-_{ j}}}{2m} +\frac{1}{2}\Bigg ], \end{aligned} \end{aligned}$$

which proves Eq. (6).

### Proof of Eq. (5)

Recall that $\mathcal {N}$ is a random element of the set of all orderings *S*(*M*). Let us define

12 $$\begin{aligned} f\!\left( \mathcal {N}\right) := \prod _{r=0}^{m-1} \frac{(m-r)^2 - \psi _r}{(m-r)^2 - \Psi _r}. \end{aligned}$$

Then Eq. (5) becomes equivalent to

13 $$\begin{aligned} \mathbb {E}\left[ f\!\left( \mathcal {N}\right) \right] = 1 + o(1), \end{aligned}$$

which we will demonstrate in the remainder of this section. We start by rewriting the latter expectation as a sum of expected values

14 $$\begin{aligned} \mathbb {E}\left[ f\!\left( \mathcal {N}\right) \right]= & {} \mathbb {E}\left[ f\!\left( \mathcal {N}\right) \mathbb {1}_{\mathcal {A}}\right] + \mathbb {E}\left[ f\!\left( \mathcal {N}\right) \mathbb {1}_{\mathcal {B}}\right] \nonumber \\{} & {} + \mathbb {E}\left[ f\!\left( \mathcal {N}\right) \mathbb {1}_{\mathcal {C}}\right] + \mathbb {E}\left[ f\!\left( \mathcal {N}\right) \mathbb {1}_{S\left( \mathcal {M}\right) \setminus S^*\left( \mathcal {M}\right) }\right] , \end{aligned}$$

of mutually disjoint subsets covering $S\left( \mathcal {M}\right)$ in the following fashion.

**Partitioning** $S\left( \mathcal {M}\right)$ The set of orderings $S\left( \mathcal {M}\right)$ is partitioned as follows:

1. For a small number $0< \tau \le \frac{1}{3}$, such that $d_{\max } = \mathcal {O}\left( m^{1/4 - \tau }\right)$, we define
  15 $$\begin{aligned} S^*\left( \mathcal {M}\right) := \left\{ \mathcal {N} \in S\left( \mathcal {M}\right) \vert \Psi _r\left( \mathcal {N}\right) - \psi _r \le \left( 1- \frac{\tau }{4}\right) \left( m-r\right) ^2, \forall \, 0\le r \le m-1\right\} , \end{aligned}$$
  and let $S\left( \mathcal {M}\right) {\setminus } S^*\left( \mathcal {M}\right)$ be the first element of the partition.
2. As the second element of the partition, we take
  16 $$\begin{aligned} \mathcal {A} := \left\{ \mathcal {N} \in S^*\left( \mathcal {M}\right) | \Psi _r\left( \mathcal {N}\right) - \psi _r \ge T_r \left( (\ln n)^{1+\delta }\right) , \exists \, 0\le r \le m-1\right\} , \end{aligned}$$
  where the family of functions $T_r$ is defined below, see Eq. (24), and $\delta$ is a small positive constant, e.g. $0< \delta < 0.1$.
3. The next element of the partition is chosen from $S^*\left( \mathcal {M}\right) \setminus \mathcal {A}$ to be
  17 $$\begin{aligned} \mathcal {B} \!:=\! \left\{ \mathcal {N} \!\in S^*\left( \mathcal {M}\right) \!\setminus \! \mathcal {A} \vert \exists 0 \!\le \! r \le m-1, \, \text {s.t.} \, m\!-\!r \!\le \! (\ln n)^{1+2\delta } \, \text {and} \, \Psi _r\left( \mathcal {N}\right) \!>\! 1\right\} . \end{aligned}$$
4. We define as last element as the complement
  18 $$\begin{aligned} \mathcal {C} := S^*\left( \mathcal {M}\right) \setminus \left( \mathcal {A} \cup \mathcal {B}\right) . \end{aligned}$$

We will now show that the following asymptotic estimates hold for the terms in Eq. (14),

19 $$\begin{aligned} \mathbb {E}\left( f\!\left( \mathcal {N}\right) \mathbb {1}_{\mathcal {A}}\right) = o(1); \end{aligned}$$

20 $$\begin{aligned} \mathbb {E}\left( f\!\left( \mathcal {N}\right) \mathbb {1}_{\mathcal {B}}\right) = o(1); \end{aligned}$$

21 $$\begin{aligned} \mathbb {E}\left( f\!\left( \mathcal {N}\right) \mathbb {1}_{\mathcal {C}}\right) \le 1+ o(1); \end{aligned}$$

22 $$\begin{aligned} \mathbb {E}\left( f\!\left( \mathcal {N}\right) \mathbb {1}_{\mathcal {C}}\right) \ge 1- o(1); \end{aligned}$$

23 $$\begin{aligned} \mathbb {E}\left( f\!\left( \mathcal {N}\right) \mathbb {1}_{S\left( \mathcal {M}\right) \setminus S^*\left( \mathcal {M}\right) }\right) = o(1). \end{aligned}$$

Hence, to finish the proof of (13), it remains to introduce suitable $T_r$ and prove Eqs. (19)–(23).

**The family of functions** $T_r$. We define the family of functions $T_r: \mathbb {R}_{\ge 0} \rightarrow \mathbb {R}_{\ge 0}$ indexed by $r \in \{0,1,\ldots , m-1\}$ as follows

24 $$\begin{aligned} \text {T}_r\left( \lambda \right) := {\left\{ \begin{array}{ll} 4\beta _r\left( \lambda \right) + 2 \min \left( \gamma _r(\lambda ), \nu _r\right) &{} \text {if }\, m -r \ge \lambda \omega ,\\ \frac{\lambda ^2}{\omega ^2}, &{} \text {otherwise}, \end{array}\right. } \end{aligned}$$

with

25 $$\begin{aligned}&\beta _r\left( \lambda \right) := c \sqrt{\lambda \left( md_{\max }^2q_r^2 + \lambda ^2\right) \left( d_{\max }^2 q_r + \lambda \right) }, \end{aligned}$$

26 $$\begin{aligned}&\gamma _r\left( \lambda \right) := c \sqrt{\lambda \left( md_{\max }^2q_r^3 + \lambda ^3\right) \left( d_{\max }^2 q_r^2 + \lambda ^2\right) }, \end{aligned}$$

27 $$\begin{aligned}&\nu _r:= 8md_{\max }^2q_r^3, \end{aligned}$$

28 $$\begin{aligned}&\omega := (\ln n)^\delta , \end{aligned}$$

29 $$\begin{aligned}&q_r := \frac{m-r}{m}=1-p_r. \end{aligned}$$

The quantity *c* is a large enough positive constant, which will bound from below in the proof of Lemma 3.13, and $q_r$ is the probability that an edge of $G_{\textbf{d}}$ is not present in $G_{p_r}$. The intuition behind the definition of this family of functions will become apparent in the remainder of this section. Let $\lambda _0 := \omega \ln n$ and $\lambda _i := 2^i\lambda _0$ for all $i \in \{1,2,\ldots , L\}$, where *L* is the smallest integer such that $\lambda _{L} \ge c\,d_{\max } \ln n$. We have the following relation between $T_r\left( \lambda _i\right)$ and $T_r\left( \lambda _{i-1}\right)$.

#### Lemma 3.6

For all $0 \le r \le m-1$ and $i \in \{1,2,\ldots , L\}$,

$$\begin{aligned} T_r\left( \lambda _{i}\right) \le 8T_r\left( \lambda _{i-1}\right) . \end{aligned}$$

#### Proof

As the function $T_r$ is defined piecewise, we distinguish three cases:

1. Suppose $$m-r < \lambda _i\omega $$ and $$ m-r < \lambda _{i-1}\omega $$.
  Then
  $$\begin{aligned} T_r(\lambda _{i}) = \frac{\lambda _i^2}{\omega ^2} = \frac{4\lambda _{i-1}^2}{\omega ^2} < \frac{8\lambda _{i-1}^2}{\omega ^2} = 8T_r(\lambda _{i-1}), \end{aligned}$$
  showing that $$ T_r\left( \lambda _{i}\right) \le 8T_r\left( \lambda _{i-1}\right) $$.
2. Suppose $$m-r < \lambda _i\omega $$ and $$ m-r \ge \lambda _{i-1}\omega $$.
  Then by definition, $T_r\left( \lambda _i\right) = \frac{4\lambda _{i-1}^2}{\omega ^2}$ and $T_r\left( \lambda _{i-1}\right) \ge 4\beta _r\left( \lambda _{i-1}\right) \ge 4c\lambda _{i-1}^2$. Hence we find $T_r\left( \lambda _i\right) \le T_r\left( \lambda _{i-1}\right)$.
3. Suppose $$m-r \ge \lambda _i\omega $$ and $$ m-r \ge \lambda _{i-1}\omega $$.
  Then by definition, $T_r\left( \lambda _i\right) = 4\beta _r\left( \lambda _i\right) + 2 \min \left( \gamma _r(\lambda _i), \nu _r\right)$ and $T_r\left( \lambda _{i-1}\right) = 4\beta _r\left( \lambda _{i-1}\right) + 2 \min \left( \gamma _r(\lambda _{i-1}), \nu _r\right)$. Both $\beta _r\left( \lambda \right)$ and $\gamma _r\left( \lambda \right)$ are square roots of a $6^\text {th}$-order polynomial in $\lambda$ with non-negative coefficients. As $\lambda _i = 2\lambda _{i-1}$ and $\sqrt{2^6} = 8$, this implies that $\beta _r\left( \lambda _{i}\right) \le 8 \beta _r\left( \lambda _{i-1}\right)$ and $\gamma _r\left( \lambda _{i}\right) \le 8 \gamma _r\left( \lambda _{i-1}\right)$. Hence, $T_r\left( \lambda _i\right) \le 8T_r\left( \lambda _{i-1}\right)$.

This completes the proof, because $m-r \ge \lambda _i\omega$ and $m-r < \lambda _{i-1}\omega$ never holds, as $\lambda _i > \lambda _{i-1}$. $\square$

To prove Eqs. (19) and (20), we subpartition $\mathcal {A}$ and $\mathcal {B}$. Let us define the chain of subsets $A_0 \subset A_1 \subset \ldots \subset A_L \subset S^*\left( \mathcal {M}\right)$ with

30 $$\begin{aligned} A_i := \left\{ \mathcal {N} \in S^*\left( \mathcal {M}\right) |\, \Psi _r\left( \mathcal {N}\right) - \psi _r < T_r\left( \lambda _i \right) , \forall 0 \le r \le m-1 \right\} . \end{aligned}$$

To ensure that we cover $S^*\left( \mathcal {M}\right)$ entirely, we also introduce

31 $$\begin{aligned}&A_{\infty } := S^*\left( \mathcal {M}\right) \setminus A_L = \{ \mathcal {N} \in S^*\left( \mathcal {M}\right) |\, \exists \, 0\nonumber \\&\quad \le r \le m-1, \,\text {s.t.}\, \Psi _r\left( \mathcal {N}\right) - \psi _r \ge T_r\left( \lambda _L\right) \}. \end{aligned}$$

Now Eq. (16) implies that

$$\begin{aligned} \mathcal {A} = S^*\left( \mathcal {M}\right) \setminus A_0 = \cup _{i=1}^L \left( A_i \setminus A_{i-1} \right) \bigcup A_{\infty }. \end{aligned}$$

Next, we partition $A_0$. The goal of this partition is to write $\mathcal {B}$ as the union of some smaller sets. As $\mathcal {N} \in A_0$ for all $0\le r\le m-1$ such that $r\ge m-\omega \lambda _0$,

$$\begin{aligned} \Psi _r\left( \mathcal {N}\right) < T_r(\lambda _0) + \psi _r = (\ln n)^{2} + \psi _r. \end{aligned}$$

According to Lemma 3.5 for all $m-1 \ge r \ge m-\omega \lambda _0$, $\psi _r = o(1)$. (Since isolated vertices are not allowed, $m-1 \ge \frac{n}{2}-1> \omega \lambda _0$ for sufficiently large *n*.) Hence, there is some $n_0$ such that for all $n > n_0$:

$$\begin{aligned} \Psi _r\left( \mathcal {N}\right) < (\ln n)^{2} + 1. \end{aligned}$$

Without loss of generality, we may assume that $n > n_0$. Let *K* be the unique integer such that

32 $$\begin{aligned} 2^{K-1} < (\ln n)^{2} + 1 \le 2^K. \end{aligned}$$

Then for all $r \ge m-\omega \lambda _0$:

$$\begin{aligned} \Psi _r\left( \mathcal {N}\right) \le 2^K. \end{aligned}$$

This allows us to define the chain of subsets $B_0 \subset B_1 \subset \ldots \subset B_K = A_0$, with

33 $$\begin{aligned} B_j = \left\{ \mathcal {N} \in A_0 |\, \Psi _r\left( \mathcal {N}\right) < 2^j, \forall r \ge m-\omega \lambda _0 \right\} . \end{aligned}$$

From Eqs. (17) and (18), it immediately follows that

$$\begin{aligned} \mathcal {B} = \cup _{i=1}^K B_i\setminus B_{i-1} \quad \text {and} \quad \mathcal {C} = B_0. \end{aligned}$$

These descriptions of $\mathcal {A}, \mathcal {B}$ and $\mathcal {C}$ enable us to show the validity of Eqs. (19), (20), (21) and (22). First, we prove Eq. (19). The proof also contains statements that hold for any ordering in $S^*\left( \mathcal {M}\right)$, which are also used in the proof of Eqs. (20), (21) and (22). We finish with the proof of Eq. (23), which requires a different technique as it concerns all orderings not in $S^*\left( \mathcal {M}\right)$.

**Proof of Equation** (19) Based on the definition of $\mathcal {A}$ in terms of $A_i$’s and $A_{\infty }$, we now prove Eq. (19). For this, we use the following Lemmas.

#### Lemma 3.7

For all $$ 1\le i \le L$$,

- $$\mathbb {P}\left[ \mathcal {N} \in A_i {\setminus } A_{i-1}\right] \le e^{-\Omega \left( \lambda _i\right) }$$;
- For all $$\mathcal {N} \in A_i\setminus A_{i-1}$$, $$f(\mathcal {N}) \le e^{o\left( \lambda _i\right) }$$.

#### Lemma 3.8

For a large enough constant *c*,

- $$\mathbb {P}\left[ \mathcal {N} \in A_{\infty }\right] \le e^{-\Omega \left( cd_{\max } \ln n \right) }$$;
- For all $$\mathcal {N} \in A_{\infty }$$, $$f(\mathcal {N}) \le e^{ 72d_{\max } \ln n}$$.

Together these lemmas imply that

$$\begin{aligned} \mathbb {E}\left[ f\!\left( \mathcal {N}\right) \mathbb {1}_{\mathcal {A}}\right]&\le \sum _{i=1}^L e^{-\Omega \left( \lambda _i\right) } e^{o\left( \lambda _i\right) } + e^{-\Omega \left( cd_{\max } \ln n\right) }e^{72d_{\max } \ln n} = o(1), \end{aligned}$$

thus proving Eq. (19).

First, we prove Lemma 3.7 (*a*) and Lemma 3.8 (*a*). This is done by showing a stronger statement,

$$\begin{aligned} \mathbb {P}\left[ \mathcal {N} \in A_{i-1}^c\right] \le e^{-\Omega \left( \lambda _i\right) }, \end{aligned}$$

for all $i \in \{0,1,\ldots , L\}$. This statement is indeed stronger than the statements of Lemma 3.7 (*a*) as $\left( A_i {\setminus } A_{i-1} \right) \subset \left( S\left( \mathcal {M}\right) {\setminus } A_{i-1}\right)$. This observation is also relevant for Lemma 3.8 (*a*), since $A_{\infty } \in A_{L}^c$ and $\lambda _L \ge cd_{\max }\ln n$. Combining the definition of $A_{i-1}$ with Lemma 3.6, we find

$$\begin{aligned} A_{i-1}^c \subset \left\{ \mathcal {N} \in S\left( \mathcal {M}\right) | \exists \, 0 \le r \le m-1 \,\text {s.t.}\, \Psi _r\left( \mathcal {N}\right) - \psi _r > \frac{T_r\left( \lambda _i\right) }{8}\right\} . \end{aligned}$$

This implies that to prove Lemma 3.7 (a) and Lemma 3.8 (a), it suffices to show that for all $i \in \{0,1,\ldots , L\}$ and $0 \le r \le m-1$,

34 $$\begin{aligned} \mathbb {P}\left[ \left|\Psi _r - \psi _r \right|\ge \frac{T_r\left( \lambda _i\right) }{8} \right] \le e^{-\Omega \left( \lambda _i\right) }. \end{aligned}$$

Determining the expected value of $\Psi _r$ as given by Algorithm 1 at step *r* is more challenging than the expectation of the same observation in random graph model $G_{p_r}$ where each edge is present with probability $p_r$. We refer to the latter expected value as $\Psi _{p_r}$. As mentioned in Sect. 3.1, the graph $G_{\mathcal {N}_r}$ is a random subgraph of $G_{\textbf{d}}$ with exactly *r* edges for a random ordering $\mathcal {N} \in S\left( \mathcal {M}\right)$. Denoting the number of edges in $G_{p_r}$ by $E\left[ G_{p_r}\right]$, we find:

$$\begin{aligned}&\mathbb {P}\left[ \left|\Psi _r - \psi _r \right|\ge \frac{T_r\left( \lambda _i\right) }{8}\right] = \frac{\mathbb {P}\left[ \left|\Psi _{p_r} - \psi _r \right|\ge \frac{T_r\left( \lambda _i\right) }{8} \cap \left|E\left[ G_{p_r}\right] \right|= r\right] }{\mathbb {P}\left[ \left|E\left[ G_{p_r}\right] \right|= r\right] } \\&\quad \le \frac{\mathbb {P}\left[ \left|\Psi _{p_r} - \psi _r \right|\ge \frac{T_r\left( \lambda _i\right) }{8} \right] }{\mathbb {P}\left[ \left|E\left[ G_{p_r}\right] \right|= r\right] } . \end{aligned}$$

Bayati, Kim, and Saberi showed the following bound on the probability that the random graph $G_{p_r}$ contains exactly *r* edges.

#### Lemma 3.9

([3, Lemma 21]) For all $0 \le r \le m$, $\mathbb {P}\left[ \left|E\left[ G_{p_r}\right] \right|= r\right] \ge \frac{1}{n}$.

Using this Lemma, we obtain

$$\begin{aligned} \mathbb {P}\left[ \left|\Psi _r - \psi _r \right|\ge \frac{T_r\left( \lambda _i\right) }{8} \right] \le n\cdot \mathbb {P}\left[ \left|\Psi _{p_r} - \psi _r \right|\ge \frac{T_r\left( \lambda _i\right) }{8} \right] . \end{aligned}$$

As $\lambda _i = 2^i (\ln n)^{1+\delta } \gg \ln n$, $ne^{-\Omega \left( \lambda _i\right) } = e^{-\Omega \left( \lambda _i\right) + \ln n} = e^{-\Omega \left( \lambda _i\right) }$. Hence, to prove Eq. (34) it suffices to show that

$$\begin{aligned} \mathbb {P}\left[ \left|\Psi _{p_r} - \psi _r \right|\ge \frac{T_r\left( \lambda _i\right) }{8} \right] \le e^{-\Omega \left( \lambda _i\right) }. \end{aligned}$$

As $T_r$ is defined piecewise, we formulate separate Lemmas distinguishing two cases:

(*i*) $$m-r < \omega \lambda _i$$ and (*ii*) $$m-r \ge \omega \lambda _i$$.

#### Lemma 3.10

For all $i \in \{0,1,\ldots , L\}$ and $0 \le r \le m-1$ such that $m-r < \lambda _i\omega$,

35 $$\begin{aligned} \mathbb {P}\left[ \Psi _{p_r} - \psi _r \ge \frac{\lambda _i^2}{8\omega ^2}\right] \le e^{-\Omega \left( \lambda _i\right) }. \end{aligned}$$

#### Proof

Instead of showing the desired inequality, we show an even stronger statement:

$$\begin{aligned} \mathbb {P}\left[ \Psi _{p_r} \ge \frac{\lambda _i^2}{8\omega ^2}\right] \le e^{-\Omega \left( \lambda _i\right) }. \end{aligned}$$

Combining the fact that $\Psi _{p_r} \le \frac{\lambda _i^2}{8\omega }$ with $\Psi _{p_r} = \Delta _{p_r} + \Lambda _{p_r}$ and Lemma 3.2(iii), we find

$$\begin{aligned} \Delta _{p_r}&\ge \frac{\lambda _i^2}{8\omega ^2} - \frac{d_{\max }^2m}{2}q_r^2. \end{aligned}$$

As $mq_r = m-r < \omega \lambda _i$ and $\omega ^4 d_{\max }^2 < \frac{m}{5}$ for large *n* we have

$$\begin{aligned} \Delta _{p_r}&\ge \frac{\lambda _i^2}{8\omega ^2} - \frac{d_{\max }^2}{2m}\omega ^2\lambda _i^2 \quad \ge \frac{\lambda _i^2}{40\omega ^2}. \end{aligned}$$

Let $G_{q_r}$ be the complement of $G_{p_r}$ in $G_{\textbf{d}}$ and define $N_0(u) := \{ v \in V \mid (u,v) \in G_{q_r}\} \cup \{u\}$. Let $d_{G_{q_r}}^+(u)$ (respectively $d_{G_{q_r}}^-(u)$) be the out-degree (in-degree) of *u* in $G_{q_r}$. By definition of $\Delta _{p_r}$,

$$\begin{aligned} \Delta _{p_r} \le \sum _{u \in V} d^+_{G_{q_r}}(u) \sum _{v \in N_0(u)}d^-_{G_{q_r}}(v). \end{aligned}$$

By combining the latter inequality with the lower bound on $\Delta _{p_r}$ we have just derived, we find

36 $$\begin{aligned} \frac{\lambda _i^2}{40\omega ^2} \le \Delta _{p_r} \le \sum _{u \in V} d^+_{G_q}(u) \sum _{v \in N_0(u)}d^-_{G_q}(v). \end{aligned}$$

This equation implies that at least one of the following statements must hold true:

- $G_q$ has more than $\frac{\omega ^2\lambda _i}{40}$ edges;
- For some $$u \in V$$, $$\sum _{v \in N_0(u)} d^-_{G_q}(v) \ge \frac{\lambda _i}{\omega ^4}$$.

If (*a*) is violated, then $\sum _{u \in V} d^+_{G_q}(u) \le \frac{\omega ^2\lambda _i}{40}$. If (*b*) is violated, $\sum _{v \in N_0(u)} d^-_{G_q}(v) < \frac{\lambda _i}{\omega ^4}$ for all $u \in V$. Hence, if (*a*) and (*b*) are both violated, we find

$$\begin{aligned} \Delta _{p_r} \le \sum _{u \in V} d^+_{G_q}(u) \sum _{v \in N_0(u)}d^-_{G_q}(v) < \frac{\omega ^2\lambda _i}{40} \frac{\lambda _i}{\omega ^4} = \frac{\lambda _i^2}{40\omega ^2}. \end{aligned}$$

This violates Eq. (36). Thus, it is not possible that (*a*) and (*b*) are simultaneously violated. This implies that at least one of the statements holds. By identical argument as in the proof of [3, Lemma 20], probabilities of both (*a*) and (*b*) are upper bounded by $e^{-\Omega \left( \lambda _i\right) }$. Indeed,

$$\begin{aligned} \mathbb {P}\left[ G_q \text { has more edges than } \frac{\omega ^2\lambda _i}{40}\right] \le \left( {\begin{array}{c}m\\ \frac{\omega ^2\lambda _i}{40}\end{array}}\right) q^{\frac{\omega ^2\lambda _i}{40}}\le e^{-\Omega \left( \lambda _i\right) }. \end{aligned}$$

Furthermore, if (b) holds, then the number of edges in the sum is at most $d_\text {max}^2$ with each edge contributing at most two times. Hence,

$$\begin{aligned} \mathbb {P}\left[ \sum _{v \in N_0(u)} d^-_{G_q}(v) \ge \frac{\lambda _i}{\omega ^4}\right] \le \left( {\begin{array}{c}d_\text {max}^2\\ \frac{\lambda _i}{\omega ^4}\end{array}}\right) q^{\frac{\lambda _i}{\omega ^4}} \le e^{-\Omega \left( \lambda _i\right) }. \end{aligned}$$

Since $\Psi _{p_r} \ge \frac{\lambda _i^2}{8\omega }$ implies that at least one of these statements holds, this completes the proof. $\square$

#### Lemma 3.11

For all $i \in \{0,1,\ldots , L\}$ and *r* such that $m-r \ge \lambda _i\omega$,

37 $$\begin{aligned} \mathbb {P}\left[ \left|\Psi _{p_r} - \psi _r \right|\ge \frac{4\beta _r(\lambda _i) + 2\min (\nu _r, \gamma _r(\lambda _i))}{8}\right] \le e^{-\Omega \left( \lambda _i\right) }. \end{aligned}$$

Recall that $\Psi _{p_r} = \Delta _{p_r}^1 + \Delta _{p_r}^2 + \frac{{\Lambda _{p_r}^1}^+{\Lambda _{p_r}^1}^- - \Lambda _{p_r}^2}{2\,m} - \frac{\Lambda _{p_r}^3}{2\,m}$, and that $\psi _r$ equals $\mathbb {E}\left[ \Psi _{p_r}\right]$. Thus, to prove Lemma 3.11, it suffices to concentrate $\Delta _{p_r}^1, \Delta _{p_r}^2 ,{\Lambda _{p_r}^1}^+{\Lambda _{p_r}^1}^-, \Lambda _{p_r}^2$ and $\Lambda _{p_r}^3$ around their expected values with probability $e^{-\Omega \left( \lambda _i\right) }$ such that the difference between their sum and the sum of their expected values is smaller than $\frac{4\beta _r(\lambda _i) + 2\min (\nu _r, \gamma _r(\lambda _i))}{8}$. This is shown using Vu’s concentration inequality.

#### Theorem 3.12

(Vu’s concentration inequality [42]). Consider independent random variables $t_1,t_2,\ldots , t_n$ with arbitrary distribution in [0, 1]. Let $Y\left( t_1,t_2,\ldots , t_n\right)$ be a polynomial of degree *k* with coefficients in (0, 1]. For any multi-set *A*, let $\partial _AY$ denote the partial derivative with respect to the variables in *A*. Define $\mathbb {E}_j(Y) = \max _{|A| \ge j} \mathbb {E}\left( \partial _A Y\right)$ for all $0 \le j \le k$. Recursively define $c_1=1, d_1=2, c_k = 2\sqrt{k}\left( c_{k-1}+1\right) , d_k = 2\left( d_{k-1} +1 \right)$. Then for any $\mathcal {E}_0> \mathcal {E}_1> \ldots > \mathcal {E}_k =1$ and $\lambda$ fulfilling

(i) $$\mathcal {E}_j \ge \mathbb {E}_j\left( Y\right) $$;
(ii) $\frac{\mathcal {E}_j}{\mathcal {E}_{j-1}} \ge \lambda + 4j\ln n$ for all $0 \le j\le k-1;$

it holds that

$$\begin{aligned} \mathbb {P}\left[ \left|Y - \mathbb {E}\left[ Y\right] \right|\ge c_k\sqrt{\lambda \mathcal {E}_0\mathcal {E}_1}\right] \le d_k e^{-\lambda /4}. \end{aligned}$$

#### Lemma 3.13

For all $i \in \{0,1,\ldots , L\}$ and $0 \le r \le m-1$,

(i) $$\mathbb {P}\left[ \left|\Delta _{p_r}^1 - \mathbb {E}\left[ \Delta _{p_r}^1\right] \right|\ge \frac{\beta _r(\lambda _i)}{8}\right] \le e^{-\Omega (\lambda _i)}$$;
(ii) $$\mathbb {P}\left[ \left|\Delta _{p_r}^2 - \mathbb {E}\left[ \Delta _{p_r}^2\right] \right|\ge \frac{\min \left( \beta _r(\lambda _i) + \gamma _r(\lambda _i), \beta _r(\lambda _i) + \nu _r\right) }{8}\right] \le e^{-\Omega (\lambda _i)}$$;
(iii) $$\mathbb {P}\left[ \left|\frac{ {\Lambda _{p_r}^1}^-{\Lambda _{p_r}^1}^+ - \Lambda _{p_r}^2}{2\,m} - \frac{ \mathbb {E}\left[ {\Lambda _{p_r}^1}^-{\Lambda _{p_r}^1}^+ - \Lambda _{p_r}^2\right] }{2\,m} \right|\ge \frac{\beta _r(\lambda _i)}{8}\right] \le e^{-\Omega (\lambda _i)}$$;
(iv) $$\mathbb {P}\left[ \left|\frac{ \Lambda _{p_r}^3}{2\,m} - \frac{ \mathbb {E}\left[ \Lambda _{p_r}^3\right] }{2\,m} \right|\ge \frac{\min \left( \beta _r(\lambda _i) + \gamma _r(\lambda _i), \beta _r(\lambda _i) + \nu _r\right) }{8}\right] \le e^{-\Omega (\lambda _i)}$$.

#### Proof

To prove each of the above equations, we write the quantity as a polynomial and apply Theorem 3.12 to it. This polynomial will be a function of *m* Bernoulli variables. Each variable $t_e$ represents an edge $e \in G_{\textbf{d}}$, that is if $e \in G_{p_r}$, then $t_e=0$ and if $e \notin G_{p_r}$, $t_e = 1$. Remark that by definition of $G_{p_r}$, see Sect. 3.1, $\mathbb {E}\left[ t_e\right] = q_r$ for all *e*. Also by definition of $G_{p_r}$, variables $t_e$ are independent of each other.

(i) Recall that $\Delta _{p_r}^{1}$ counts the number of pairs creating a self-loop. Each vertex *v* has ${d^-_{ v}}$ in-stubs and ${d^+_{v}}$ out-stubs. The number of those out-stubs (respectively in-stubs) that are matched equals the number of outgoing (incoming edges) for *v* in $G_{p_r}$. Thus, the number of unmatched in-stubs (respectively out-stubs) of vertex *v* is $\sum _{e = (\bullet , v) \in G_{\textbf{d}}} t_e$ $\left( \sum _{e = (v, \bullet ) \in G_{\textbf{d}}} t_e \right)$. The number of ways to create a self-loop at *v* is
  $$\begin{aligned} \sum _{e=(v,\bullet ) \in G_{\textbf{d}}} \sum _{f=(\bullet ,v) \in G_{\textbf{d}}} t_et_f. \end{aligned}$$
  Hence, we find
  38 $$\begin{aligned} \Delta _{p_r}^1 = \sum _{ v \in V} \sum _{e=(v,\bullet ) \in G_{\textbf{d}}} \sum _{f=(\bullet ,v) \in G_{\textbf{d}}} t_et_f. \end{aligned}$$
  Vu’s concentration inequality requires us to upper bound the values $\mathbb {E}_0 \left[ \Delta _{p_r}^1\right] ,\mathbb {E}_1 \left[ \Delta _{p_r}^1\right]$ and $\mathbb {E}_2 \left[ \Delta _{p_r}^1\right]$. Let us first consider the expectation of $\Delta _{p_r}^1$. Because $G_{\textbf{d}}$ is simple, for each element of the summation in Eq. (38), *e* does not equal *f*. Therefore, $\mathbb {E}[t_et_f] = q_r^2$. The summations over *v* and *e* in Eq. (38) can be replaced by one summation over all edges in $G_{\textbf{d}}$. For each edge $e \in G_{\textbf{d}}$, there are at most $d_{\max }$ edges in $G_{\textbf{d}}$ with the source of *e* as target. Hence, we find $\mathbb {E}\left[ \Delta _{p_r}^1\right] \le md_{\max }q_r^2$. Let us take the partial derivative with respect to one variable $t_e$ for some $e= (u,v)$, then we obtain $\sum _{f = (\bullet , u) \in G_{\textbf{d}}} t_f + \sum _{f = (v,\bullet ) \in G_{\textbf{d}}}t_f$. This is upper bounded by $2d_{\max }q_r$. As $\Delta _{p_r}^1$ is a polynomial of degree 2 with all coefficients 1, it is clear that $\mathbb {E}\left[ \partial _{t_e}\partial _{t_f} \Delta _{p_r}^1 \right] \le 1$ for all *e*, *f*. Thus, we find
  $$\begin{aligned}&\mathbb {E}_0\left[ \Delta _{p_r}^1\right] \le \max \left( 1,2d_{\max }q_r, md_{\max }q_r^2\right) , \; \mathbb {E}_1\left[ \Delta _{p_r}^1\right] \\&\quad \le \max \left( 1, 2d_{\max }q_r\right) , \; \text {and}\; \mathbb {E}_2\left[ \Delta _{p_r}^1\right] \le 1. \end{aligned}$$
  The maximisation follows from the definition of $\mathbb {E}_j(Y)$. Let us define,
  $$\begin{aligned} \mathcal {E}_0 := 9\lambda _i^2 + 2md_{\max }q_r^2 ,\quad \mathcal {E}_1 := 9\lambda _i + 2d_{\max }q_r\quad \text {and}\quad \mathcal {E}_2 :=1. \end{aligned}$$
  We claim that together with $\lambda = \lambda _i$, they fulfil the conditions of Theorem 3.12. It is obvious that $\mathcal {E}_2 \ge \mathbb {E}_2\left[ \Delta _{p_r}^1\right]$. Also $\mathcal {E}_1 \ge \mathbb {E}_1\left[ \Delta _{p_r}^1\right]$ as $\lambda _i \ge 1$ for all $n \ge 3$. Furthermore, $\mathcal {E}_0 \ge \mathbb {E}_0\left[ \Delta _{p_r}^1\right]$ as $\lambda _i \ge 1$ and $mq_r= m-r$ implies that $2md_{\max }q_r^2 \ge 2d_{\max }q_r$. This shows the first condition of Theorem 3.12. For the second condition, remark that $\lambda _i \ge \ln n$ and $\ln (m) \le 2\ln n$ as $m \le n^2$. This implies
  $$\begin{aligned} \frac{\mathcal {E}_1}{\mathcal {E}_2} = \mathcal {E}_1 \ge \lambda _i + 4\ln (m). \end{aligned}$$
  Furthermore,
  $$\begin{aligned} \frac{\mathcal {E}_0}{\mathcal {E}_1} = \lambda _i \left( \frac{9\lambda _i + \frac{2d_{\max }mq_r^2}{\lambda _i}}{9 + \frac{2d_{\max }q_r}{\lambda _i}}\right) \ge \lambda _i, \end{aligned}$$
  showing that the second condition of Theorem 3.12 is fulfilled as well. Thus, we may apply Vu’s concentration inequality to obtain
  $$\begin{aligned} \mathbb {P}\left[ \left|\Delta _{p_r}^1 - \mathbb {E}\left[ \Delta _{p_r}^1\right] \right|\ge c_2\sqrt{\lambda _i\left( 9\lambda _i + 2d_{\max }q_r\right) \left( 9\lambda _i^2 + 2md_{\max }q_r^2\right) }\right] \le e^{-\Omega (\lambda _i)}. \end{aligned}$$
  Since $\mathbb {P}\left[ \left|\Delta _{p_r}^1 - \mathbb {E}\left[ \Delta _{p_r}^1\right] \right|\ge a\right] \le \mathbb {P}\left[ \left|\Delta _{p_r}^1 - \mathbb {E}\left[ \Delta _{p_r}^1\right] \right|\ge b\right]$ for $a >b$, choosing any $c>8\cdot 9c_2$ in Eq. (25) completes the proof.
(ii) Recall that $\Delta _{p_r}^{2}$ counts the number of pairs that create an edge already present in $G_{p_r}$, *i.e.* a double edge. Pairing an out-stub of *u* with an in-stub of *v* creates a double edge only if $(u,v) \in G_{p_r}$, i.e., if for $e = (u,v)$, $t_e = 1$. Recalling the expressions for the number of unmatched in-stubs and out-stubs at a vertex *v* from the proof of (*i*) and defining a set of non-cyclic three-edge line subgraphs,
  $$\begin{aligned}{} & {} Q = \left\{ (e,f,g)| e,f,g \in G_{\textbf{d}}, e \ne f, f\ne g, e\ne g, f = (u,v), e = (u, \bullet ), \right. \\{} & {} \quad \left. g = (\bullet , v)\right\} , \end{aligned}$$
  we find
  $$\begin{aligned} \Delta _{p_r}^2 = \sum _{ (e,f,g) \in Q} t_et_g(1-t_f) = \sum _{ (e,f,g) \in Q} t_et_g - \sum _{ e,f,g \in Q} t_et_gt_f = Y_1 - Y_2. \end{aligned}$$
  Vu’s inequality will be applied to $Y_1$ and $Y_2$ separately. To upper bound the expected value of $Y_1$, we need an upper bound on the size of *Q*. Given *f*, the source of *e* and the target of *g* are fixed. Hence, there are atmost $d_{\max }^2$ triples in *Q* with a fixed edge *f*. As *f* may be any edge, $|Q| \le md_{\max }^2$. Together with $\mathbb {E}\left[ t_et_g\right] = q_r^2$, this implies that $\mathbb {E}\left[ Y_1\right] \le md_{\max }^2q_r^2$. We differentiate $Y_1$ with respect to $t_{{\widetilde{e}}}$, to obtain:
  $$\begin{aligned} \sum _{\mathop {\begin{array}{c} (e,f,g) \in Q\\ e = {\widetilde{e}} \end{array}}} t_g \,+\, \sum _{\mathop {\begin{array}{c} (e,f,g) \in Q\\ g = {\widetilde{e}} \end{array}}} t_e. \end{aligned}$$
  Since
  $$\begin{aligned}\sum _{\mathop {\begin{array}{c} (e,f,g) \in Q\\ e = {\widetilde{e}} \end{array}}}1 \le d_{\max }^2 \quad \text {and} \quad \sum _{\mathop {\begin{array}{c} (e,f,g) \in Q\\ g = {\widetilde{e}} \end{array}}}1 \le d_{\max }^2, \end{aligned}$$
  we have $\mathbb {E}\left[ \partial _{t_{{\widetilde{e}}}}Y_1\right] \le 2d_{\max }^2q_r$, and since $Y_1$ is a polynomial of degree 2 with all coefficients equal to 1, all second derivatives are atmost 1. Together, these observations yield:
  $$\begin{aligned}&\mathbb {E}_0\left[ Y_1\right] \le \max \left( 1,2d_{\max }^2q_r,md_{\max }^2q_r^2\right) , \quad \mathbb {E}_1\left[ Y_1\right] \le \max \left( 1, 2d_{\max }^2q_r\right) \quad \text {and}\\&\quad \mathbb {E}_2\left[ Y_1\right] \le 1. \end{aligned}$$
  Similar to (*i*), it can be shown that $\lambda = \lambda _i$ and
  $$\begin{aligned} \mathcal {E}_0 = 9\lambda _i^2 + 2md_{\max }^2q_r^2 ,\quad \mathcal {E}_1 = 9\lambda _i + 2d_{\max }^2q_r\quad \text {and}\quad \mathcal {E}_2 =1, \end{aligned}$$
  fulfil the conditions of Theorem 3.12. Applying Vu’s inequality and assuming $c \ge 8\cdot 9c_2$, we thus obtain
  $$\begin{aligned} \mathbb {P}\left[ \left|Y_1- \mathbb {E}\left[ Y_1\right] \right|\ge \frac{\beta _r(\lambda _i)}{8}\right] \le e^{-\Omega (\lambda _i)}. \end{aligned}$$
  Moving on to $Y_2$, we see that $\mathbb {E}\left[ Y_2\right] \le md_{\max }^2q_r^3$ as $|Q| \le md_{\max }^2$ and $\mathbb {E}\left[ t_et_ft_g\right] = q_r^3$. Differentiating $Y_2$ to with respect $t_{{\widetilde{e}}}$, we obtain
  $$\begin{aligned} \sum _{\mathop {\begin{array}{c} (e,f,g) \in Q\\ e = {\widetilde{e}} \end{array}}} t_ft_g \,+ \, \sum _{\mathop {\begin{array}{c} (e,f,g) \in Q\\ f = {\widetilde{e}} \end{array}}} t_et_g\, +\, \sum _{\mathop {\begin{array}{c} (e,f,g) \in Q\\ g = {\widetilde{e}} \end{array}}} t_et_f. \end{aligned}$$
  This implies that $\mathbb {E}\left[ \partial _{t_{{\widetilde{e}}}}Y_1\right] \le 3d_{\max }^2q_r$. Differentiating $Y_2$ to with respect $t_{{\widetilde{e}}}$ and $t_{{\widetilde{f}}}$ for ${\widetilde{e}} \ne {\widetilde{f}}$, we obtain
  $$\begin{aligned} \sum _{\mathop {\begin{array}{c} (e,f,g) \in Q\\ e = {\widetilde{e}}\\ f = {\widetilde{f}}\\ \end{array}}} t_g \!+ \! \sum _{\mathop {\begin{array}{c} (e,f,g) \in Q\\ e = {\widetilde{e}}\\ g = {\widetilde{f}}\\ \end{array}}} t_f \!+ \! \sum _{\mathop {\begin{array}{c} (e,f,g) \in Q\\ f = {\widetilde{e}}\\ g = {\widetilde{f}}\\ \end{array}}} t_e \!+\! \sum _{\mathop {\begin{array}{c} (e,f,g) \in Q\\ f = {\widetilde{e}}\\ e = {\widetilde{f}}\\ \end{array}}} t_g \!+ \! \sum _{\mathop {\begin{array}{c} (e,f,g) \in Q\\ g = {\widetilde{e}}\\ e = {\widetilde{f}}\\ \end{array}}} t_f \!+\! \sum _{\mathop {\begin{array}{c} (e,f,g) \in Q\\ g = {\widetilde{e}}\\ f = {\widetilde{f}}\\ \end{array}}} t_e. \end{aligned}$$
  In each of the sums, there is freedom to choose only one edge. As the source, the target or both are fixed for this edge, each summation is upper bounded by $d_{\max }q_r$. According to the definition of *Q*, at most two of the summations are non-zero, implying that $\mathbb {E}\left[ \partial _{t_{{\widetilde{e}}}}\partial _{t_{{\widetilde{f}}}}Y_2\right] \le 2d_{\max }q_r$. As $Y_2$ is a polynomial of degree 3 and all of its coefficients are 1, any third-order partial derivative of $Y_2$ can be atmost 1. We, thus, find:
  $$\begin{aligned}&\mathbb {E}_0\left[ Y_2\right] \le \max \left( 1,2d_{\max }q_r,3d_{\max }^2q_r^2,md_{\max }^2q_r^3\right) ,\\&\mathbb {E}_1\left[ Y_2\right] \le \max \left( 1, 2d_{\max }q_r, 3d_{\max }^2q_r^2\right) , \,\\ {}&\mathbb {E}_2\left[ Y_2\right] \le \max \left( 1, 2d_{\max }q_r\right) \; \text {and}\; \mathbb {E}_3\left[ Y_2\right] \le 1. \end{aligned}$$
  Vu’s inequality is applied to $Y_2$ using $\lambda = \lambda _i$ and
  $$\begin{aligned}&\mathcal {E}_0 = 85\lambda _i^3 + 3md_{\max }^2q_r^3 ,\quad \mathcal {E}_1 = 85\lambda _i^2 + 3d_{\max }^2q_r^2,\\&\quad \mathcal {E}_2 = 17\lambda _i + 2d_{\max }q_r \quad \text {and}\quad \mathcal {E}_3 =1, \end{aligned}$$
  to obtain
  $$\begin{aligned} \mathbb {P}\left[ \left|Y_2 - \mathbb {E}\left[ Y_2\right] \right|\ge 85 c_3\sqrt{\lambda _i\left( \lambda _i^2 + d_{\max }^2q_r^2\right) \left( \lambda _i^3 + md_{\max }^2q_r^3\right) }\right] \le e^{-\Omega (\lambda _i)}. \end{aligned}$$
  If we choose *c* large enough, this implies that
  $$\begin{aligned} \mathbb {P}\left[ \left|\Delta _{p_r}^2 - \mathbb {E}\left[ \Delta _{p_r}^2\right] \right|\ge \frac{\beta _r(\lambda _i) + \gamma _r(\lambda _i)}{8}\right] \le e^{-\Omega (\lambda _i)}. \end{aligned}$$
  Next, remark that
  $$\begin{aligned} \left|\Delta _{p_r}^2 - \mathbb {E}\left[ \Delta _{p_r}^2\right] \right|&= \left|Y_1 - Y_2 - \mathbb {E}\left[ Y_1\right] + \mathbb {E}\left[ Y_2\right] \right|\le \left|Y_1 - \mathbb {E}\left[ Y_1\right] \right|+ \mathbb {E}\left[ Y_2\right] \\&\le \left|Y_1 - \mathbb {E}\left[ Y_1\right] \right|+ md_{\max }^2q_r^3 = \left|Y_1 - \mathbb {E}\left[ Y_1\right] \right|+ \frac{\nu _r}{8}. \end{aligned}$$
  This implies that
  $$\begin{aligned} \mathbb {P}\left[ \left|\Delta _{p_r}^2 - \mathbb {E}\left[ \Delta _{p_r}^2\right] \right|\ge \frac{\beta _r(\lambda _i) + \nu _r}{8}\right] \le e^{-\Omega (\lambda _i)}, \end{aligned}$$
  completing the proof.
(iii) To prove that $\mathbb {P}\left[ \left|\frac{ {\Lambda _{p_r}^1}^-{\Lambda _{p_r}^1}^+ - \Lambda _{p_r}^2}{2m} - \frac{ \mathbb {E}\left[ {\Lambda _{p_r}^1}^-{\Lambda _{p_r}^1}^+ - \Lambda _{p_r}^2\right] }{2m} \right|\ge \frac{\beta _r(\lambda _i)}{8}\right] \le e^{-\Omega (\lambda _i)}$, Vu’s inequality is applied to $\frac{{\Lambda _{p_r}^1}^+{\Lambda _{p_r}^1}^-}{d_{\max }^2}$ and $\frac{\Lambda _{p_r}^2}{d_{\max }^2}$ separately. The construction is almost identical to the proofs of (*i*) and (*ii*). First consider
  $$\begin{aligned} \frac{{\Lambda _{p_r}^1}^+{\Lambda _{p_r}^1}^-}{d_{\max }^2}&= \frac{\sum _{i=1}^n{{d^-_{ i}}}^{(r)}{d^-_{ i}} \sum _{i=1}^n {{d^+_{i}}}^{(r)}{d^+_{i}}}{d_{\max }^2}\\&= \left( \sum _{e = (u,v) \in G_{\textbf{d}}} \frac{{d^-_{ u}}}{d_{\max }} t_e\right) \left( \sum _{f = (w,z) \in G_{\textbf{d}}} \frac{{d^+_{z}}}{d_{\max }} t_f\right) \\&= \left( \sum _{e = (u,v) \in G_{\textbf{d}}} \frac{d_u^-d_v^+}{d_{\max }^2} t_e^2\right) + \sum _{\mathop {\begin{array}{c} e=(u,v)\in G_{\textbf{d}}\\ f = (w,z) \in G_{\textbf{d}}\\ e \ne f \end{array}}} \frac{d_u^-d_z^+}{d_{\max }^2} t_et_f = Z_1 + Z_2. \end{aligned}$$
  Start with $Z_1$. Since for a Bernoulli variable $t_e^2 = t_e$, $Z_1$ is a polynomial of degree one. Since its coefficients are atmost 1, it is clear that any first-order partial derivative of $Z_1$ is upper bounded by 1. The expected value of $Z_1$ is upper bounded by $mq_r$. This implies that,
  $$\begin{aligned} \mathbb {E}_0\left[ Z_1\right] \le \max \left( 1, mq_r\right) \quad \text {and}\quad \mathbb {E}_1\left[ Z_1\right] \le 1. \end{aligned}$$
  Hence, $\mathcal {E}_0 = mq_r + \lambda _i \quad \text {and}\quad \mathcal {E}_1 = 1,$ satisfy the constraints of Theorem 3.12 with $\lambda = \lambda _i$. Applying this theorem, we find
  $$\begin{aligned} \mathbb {P}\left[ \left|Z_1 - \mathbb {E}\left[ Z_1\right] \right|\ge c_1 \sqrt{\lambda _i\left( \lambda _i + mq_r\right) }\right] \le e^{-\Omega \left( \lambda _i\right) }. \end{aligned}$$
  Next, consider $Z_2$. This is a sum over all pairs of distinct edges; hence, it contains fewer than $m^2$ terms. Combining this with $\frac{{d^-_{ u}}{d^+_{z}}}{d_{\max }^2} \le 1$ and $\mathbb {E}\left[ t_et_f\right] = q_r^2$, we find that $\mathbb {E}\left[ Z_2\right] \le m^2q_r^2$. Taking the partial derivative with respect to a variable $t_g$ and writing $g = (i,j)$ leads to
  $$\begin{aligned} \sum _{\mathop {\begin{array}{c} f = (w,z) \in G_{\textbf{d}}\\ f \ne g \end{array}}} \frac{{d^-_{ i}}{d^+_{z}}}{d_{\max }^2}t_f \, + \, \sum _{\mathop {\begin{array}{c} e = (u,v) \in G_{\textbf{d}}\\ e \ne g \end{array}}} \frac{{d^-_{ u}}{d^+_{j}}}{d_{\max }^2}t_e. \end{aligned}$$
  Each term of the summations is upper bounded by $q_r$. Each summation contains $m-1$ terms. Thus, we find: $\mathbb {E}\left[ \partial _{t_g}Z_2\right] \le 2mq_r$. As $Z_2$ is a second-order polynomial with coefficients upper bounded by 1, all second-order partial derivatives will be at most 1. Combining these observations, we find:
  $$\begin{aligned}&\mathbb {E}_0\left[ Z_2\right] \le \max \left( 1,2mq_r,m^2q_r^2\right) ,\\&\mathbb {E}_1\left[ Z_2\right] \le \max \left( 1, 2mq_r\right) \quad \text {and}\quad \mathbb {E}_2\left[ Z_2\right] \le 1. \end{aligned}$$
  Similar to the proof of (*i*), it can be shown that $\lambda = \lambda _i$ and
  $$\begin{aligned} \mathcal {E}_0 = 9\lambda _i^2 + 2m^2q_r^2 ,\quad \mathcal {E}_1 = 9\lambda _i + 2mq_r\quad \text {and}\quad \mathcal {E}_2 =1, \end{aligned}$$
  satisfy the constraints of Vu’s concentration inequality, which gives
  $$\begin{aligned} \mathbb {P}\left[ \left|Z_2 - \mathbb {E}\left[ Z_2\right] \right|\ge 9c_2 \sqrt{\lambda _i\left( \lambda _i + mq_r\right) \left( \lambda _i^2 + m^2q_r^2\right) }\right] \le e^{-\Omega \left( \lambda _i\right) }. \end{aligned}$$
  Since $\sqrt{\lambda _i\left( \lambda _i + mq_r\right) }\le \sqrt{\lambda _i\left( \lambda _i + mq_r\right) \left( \lambda _i^2 + m^2q_r^2\right) }$ and $\frac{{\Lambda _{p_r}^1}^+{\Lambda _{p_r}^1}^-}{2\,m}= \frac{d_{\max }^2}{2\,m}\left( Z_1 + Z_2\right)$, we obtain
  $$\begin{aligned}&\mathbb {P}\left[ \left|\frac{ {\Lambda _{p_r}^1}^-{\Lambda _{p_r}^1}^+}{2m} - \frac{ \mathbb {E}\left[ {\Lambda _{p_r}^1}^-{\Lambda _{p_r}^1}^+ \right] }{2m} \right|\right. \\&\quad \left. \ge \frac{d_{\max }^2}{2m}(9c_2 + c_1)\sqrt{\lambda _i\left( \lambda _i + mq_r\right) \left( \lambda _i^2 + m^2q_r^2\right) }\right] \\&\quad \le e^{-\Omega (\lambda _i)}. \end{aligned}$$
  Pulling the factor $\frac{d_{\max }^2}{m}$ inside the root and taking $c > 8\left( c_1 + 9c_2\right)$, we also find
  $$\begin{aligned}&\mathbb {P}\left[ \left|\frac{ {\Lambda _{p_r}^1}^-{\Lambda _{p_r}^1}^+}{2m} - \frac{ \mathbb {E}\left[ {\Lambda _{p_r}^1}^-{\Lambda _{p_r}^1}^+ \right] }{2m} \right|\ge \frac{c}{8}\sqrt{\lambda _i\left( \lambda _i + d_{\max }^2q_r\right) \left( \lambda _i^2 + md_{\max }^2q_r^2\right) }\right] \\&\quad \le e^{-\Omega (\lambda _i)}. \end{aligned}$$
  Next, we consider
  $$\begin{aligned} \frac{\Lambda _{p_r}^2}{d_{\max }^2} = \sum _{i=1}^n \frac{{{d^-_{ i}}}^{(r)}{d^-_{ i}}{{d^+_{i}}}^{(r)}{d^+_{i}}}{d_{\max }^2} = \sum _{i=1}^n \frac{{d^-_{ i}}{d^+_{i}}}{d_{\max }^2} \left( \sum _{e=(i,\bullet ) \in G_{\textbf{d}}} t_e\right) \left( \sum _{f=(\bullet ,i) \in G_{\textbf{d}}} t_f\right) . \end{aligned}$$
  Note that this is the same expression as for $\Delta _{p_r}^1$ where the coefficient of each term is replaced by $\frac{\Lambda _{p_r}^2}{d_{\max }^2}$. Hence, using the same argument as for (*i*), we obtain
  $$\begin{aligned} \mathbb {P}\left[ \left|\frac{ \Lambda _{p_r}^2}{2m} - \frac{ \mathbb {E}\left[ \Lambda _{p_r}^2\right] }{2m} \right|\ge 9c_2\frac{d_{\max }^2}{2m} \sqrt{\lambda _i\left( \lambda _i + q_rd_{\max }\right) \left( \lambda _i^2 + md_{\max }q_r^2\right) }\right] \le e^{-\Omega (\lambda _i)}. \end{aligned}$$
  Again pulling $\frac{d_{\max }^2}{m}$ inside the square root, we find
  $$\begin{aligned}&\mathbb {P}\left[ \left|\frac{ {\Lambda _{p_r}^1}^-{\Lambda _{p_r}^1}^+ - \Lambda _{p_r}^2}{2m} - \frac{ \mathbb {E}\left[ {\Lambda _{p_r}^1}^-{\Lambda _{p_r}^1}^+ - \Lambda _{p_r}^2 \right] }{2m} \right|\right. \\&\quad \left. \ge 9c_2\sqrt{\lambda _i\left( \lambda _i + d_{\max }^2q_r\right) \left( \lambda _i^2 + md_{\max }^2q_r^2\right) }\right] \\&\quad \le e^{-\Omega (\lambda _i)}. \end{aligned}$$
  Since $\beta = c\sqrt{\lambda _i\left( \lambda _i + d_{\max }^2q_r\right) \left( \lambda _i^2 + md_{\max }^2q_r^2\right) }$, this completes the proof by taking $c > 8(18c_2 + c_1)$.
(iv) This argument is exactly the same as for (*ii*), since
  $$\begin{aligned} \frac{\Lambda _{p_r}^3}{d_{\max }^2} = \sum _{\mathop {\begin{array}{c} (e,f,g) \in Q\\ e = (u,v) \end{array}}}\frac{{d^+_{u}}d_v^-}{d_{\max }^2} t_e\left( 1-t_f\right) t_g. \end{aligned}$$
  Hence, we obtain $\mathbb {P}\left[ \left|\frac{ \Lambda _{p_r}^3}{2\,m} - \frac{ \mathbb {E}\left[ \Lambda _{p_r}^3\right] }{2\,m} \right|\ge \frac{d_{\max }^2}{2\,m}\frac{\min \left( \beta _r(\lambda _i) + \gamma _r(\lambda _i), \beta _r(\lambda _i) + \nu _r\right) }{8}\right] \le e^{-\Omega (\lambda _i)},$ and since $\frac{d_{\max }^2}{m} = o(1)$, this completes the proof.

$$\square $$

Combining all inequalities from the statement of Lemma 3.13, we find that

$$\begin{aligned} \mathbb {P}\left[ \left|\Psi _{p_r} - \mathbb {E}\left[ \Psi _{p_r}\right] \right|\ge \frac{4\beta _r(\lambda _i) + 2\min (\nu _r, \gamma _r(\lambda _i))}{8}\right] \le e^{-\Omega \left( \lambda _i\right) }, \end{aligned}$$

for all $i \in \{0,1,\ldots , L\}$ and $0 \le r \le m-1$. By definition of $\psi _r$ this shows Eq. (37), and hence it proves Lemma 3.11. This completes the proofs of Lemma 3.7 (*a*) and 3.8 (*a*).

Next, we prove Lemma 3.7 (*b*) and 3.8 (*b*). This requires the following Lemma.

#### Lemma 3.14

For all $i \in \{1,2,\ldots , L\}$ and $\mathcal {N} \in A_i\setminus A_{i-1}$,

$$\begin{aligned} \sum _{r=0}^{m-1} \frac{\max \left( \Psi _r\left( \mathcal {N}\right) - \psi _r, 0 \right) }{(m-r)^2 - \Psi _r\left( \mathcal {N}\right) } = o\left( \lambda _i\right) . \end{aligned}$$

Furthermore for all $\mathcal {N} \in A_0$,

$$\begin{aligned} \sum _{m-r= \lambda _0\omega }^{m} \frac{\max \left( \Psi _r\left( \mathcal {N}\right) - \psi _r, 0 \right) }{(m-r)^2 - \Psi _r\left( \mathcal {N}\right) } = o\left( 1\right) . \end{aligned}$$

#### Proof

The first claim follows by changing the summation $\sum _{m-r=2}^{2m-2}$ into $\sum _{m-r=1}^m$ in the proof of Lemma 15(*b*) [3]. The second claim follows by applying a similar change to the proof of Lemma 18 [3]. $\square$

We will now determine an upper bound on $f\!\left( \mathcal {N}\right)$ for all $\mathcal {N} \in S^*\left( \mathcal {M}\right)$. According to the definition of $S^*\left( \mathcal {M}\right)$, $\Psi _r\left( \mathcal {N}\right) \le \left( 1- \frac{\tau }{4}\right) (m-r)^2$ holds for all $0\le r\le m-1$. Therefore,

$$\begin{aligned} f\!\left( \mathcal {N}\right)&= \prod _{r=0}^{m-1}\left( 1+\frac{\Psi _r\left( \mathcal {N}\right) - \psi _r}{(m-r)^2 - \Psi _r\left( \mathcal {N}\right) }\right) \le \prod _{r=0}^{m-1}\left( 1+\frac{4\max \left( \Psi _r\left( \mathcal {N}\right) - \psi _r, 0 \right) }{\tau (m-r)^2}\right) . \end{aligned}$$

Using $1 + x \le e^x$, the latter inequality becomes

39 $$\begin{aligned} f\!\left( \mathcal {N}\right)&\le e^{\sum _{r=0}^{m-1}\frac{4\max \left( \Psi _r\left( \mathcal {N}\right) - \psi _r, 0 \right) }{\tau (m-r)^2}}. \end{aligned}$$

Let us consider $\mathcal {N} \in A_i\setminus A_{i-1}$ for $i \in \{1,2,\ldots , L\}$ and apply Lemma 3.14 to Eq. (39), to obtain:

$$\begin{aligned} f\!\left( \mathcal {N}\right)&\le e^{o\left( \lambda _i\right) }. \end{aligned}$$

This completes the proof of Lemma 3.7 (*b*).

It remains to prove Lemma 3.8 (*b*). As $A_{\infty } \subset S^*\left( \mathcal {M}\right)$ we have, for $\mathcal {N}\in A_{\infty }$:

$$\begin{aligned} f\!\left( \mathcal {N}\right)&\le \prod _{r=0}^{m-d_{\max }^2} \left( 1+\frac{4\max \left( \Psi _r\left( \mathcal {N}\right) - \psi _r, 0 \right) }{\tau (m-r)^2}\right) \prod _{r=m-d_{\max }^2 +1 }^{m-1} \frac{(m-r)^2 - \psi _r}{(m-r)^2-\Psi _r\left( \mathcal {N}\right) }. \end{aligned}$$

Since $0 <\Psi _r\left( \mathcal {N}\right)$ and $\psi _r < (m-r)^2$, we further have:

$$\begin{aligned} f\!\left( \mathcal {N}\right)&\le \left( d_{\max }^4\right) ^{d_{\max }^2}\prod _{r=0}^{m-d_{\max }^2} \left( 1+\frac{4\Psi _r\left( \mathcal {N}\right) }{\tau (m-r)^2}\right) . \end{aligned}$$

From Lemma 3.2, it follows that $\Psi _r = \Delta _r + \Lambda _r \le 2 (m-r)d_{\max }^2$, which, when inserted in the latter inequality, gives:

$$\begin{aligned} f\!\left( \mathcal {N}\right)&\le \left( d_{\max }^4\right) ^{d_{\max }^2}\prod _{r=0}^{m-d_{max}^2} \left( 1+\frac{8d_{\max }^2}{\tau (m-r)}\right) . \end{aligned}$$

Using $(1+x) \le e^x$, we find:

$$\begin{aligned} f\!\left( \mathcal {N}\right)&\le e^{4d_{\max }^2\ln \left( d_{\max }\right) + \frac{8}{\tau } \sum _{i= d_{\max }^2}^{m}i^{-1}d_{\max }^2} \le e^{4d_{\max }^2\ln \left( d_{\max }\right) + \frac{8}{\tau } \ln (m) - \frac{8}{\tau }\ln (d_{\max }^2)}, \end{aligned}$$

and since $\tau \le \frac{1}{3}$ and $m \le nd_{\max }$, we have:

$$\begin{aligned} f\!\left( \mathcal {N}\right)&\le e^{4d_{\max }^2\ln \left( d_{\max }\right) + 24\ln (m)} \le e^{4d_{\max }^2\ln \left( d_{\max }\right) + 24\ln (nd_{\max })}\\&\le e^{24d_{\max }^2\ln \left( nd_{\max }^2\right) } \le e^{24d_{\max }^2\ln \left( n^3\right) } = e^{72d_{\max }^2\ln \left( n\right) }. \end{aligned}$$

This proves Lemma 3.8 (*b*), completes the proofs of Lemma’s 3.7 and 3.8 and, therefore, completes the proof of the asymptotic estimate (19).

**Proof of Equation** (20) The next step is showing that Eq. (20) holds. To this end, we first prove the following Lemma.

#### Lemma 3.15

For all $$ 1 \le j \le K$$

- $$\mathbb {P}\left[ \mathcal {N} \in B_j{\setminus } B_{j-1}\right] \le e^{-\Omega \left( 2^{j/2} \ln n\right) }$$;
- For all $$\mathcal {N} \in B_j\setminus B_{j-1}$$, $$f\!\left( \mathcal {N}\right) \le e^{\mathcal {O}\left( 2^j\right) }$$.

#### Proof

- The probability that $\mathcal {N}\in B_{j}\setminus B_{j-1}$ is upper bounded by the probability that
  $\mathcal {N}\in B_{j-1}^c := S\left( \mathcal {M}\right) {\setminus } B_{j-1}$. Hence, if we show that
  $$\begin{aligned} \mathbb {P}\left[ \mathcal {N} \in B_j^c\right] \le e^{-\Omega \left( 2^{j/2}\ln n\right) }, \end{aligned}$$
  the claim is proven. Remark that $B_{j-1}^c \subset \left\{ \mathcal {N} \in S\left( \mathcal {M}\right) | \,\exists \, r, \, \text {s.t.} \, m-r\right. \left. \le \omega \lambda _0 \, \text {and} \, \Psi _r \ge 2^{j-1} \right\} .$ Therefore, we need to consider only those *r* for which $m-r \le \omega \lambda _0$.
  Note that
  40 $$\begin{aligned} \mathbb {P}\left[ \Psi _r \ge 2^{j-1} | r \ge m-\omega \lambda _0 \right] \le e^{-\Omega \left( 2^{j/2}\ln n\right) } \end{aligned}$$
  is a stronger statement than the desired inequality. Indeed, using $\omega \lambda _0 \ll (\ln n)^2$ and the union bound gives:
  $$\begin{aligned} \mathbb {P}\left[ \mathcal {N} \in B_{j-1}^c\right]&\le \ln ^2(n) \mathbb {P}\left[ \Psi _r \ge 2^{j-1}\right] \le \ln ^2(n) e^{-\Omega \left( 2^{j/2}\ln n\right) } \\ {}&= e^{-\Omega \left( 2^{j/2}\ln n\right) + 2\ln \left( \ln n\right) } = e^{-\Omega \left( 2^{j/2}\ln n\right) } . \end{aligned}$$
  We will, therefore, prove inequality (40) instead. Fix an arbitrary *r* such that $m-r < \omega \lambda _0$. First, we show that $\Psi _r \le 2^{j-1}$implies that $\Delta _r \ge 2^{j-1}$ and then estimate the probability of the later event. From Lemma 3.2 and the definition of $\Psi _r$, we have
  $$\begin{aligned} \Delta _r&\ge 2^{j-1} - \frac{d_{\max }^2m}{2}q_r^2. \end{aligned}$$
  Since $$m-r \le \omega \lambda _0 < 2^{j-1}\omega \lambda _0$$ and $$d_{\max }^2\omega ^2\lambda _0^2 < m$$,
  $$\begin{aligned} \Delta _r&\ge 2^{j-1} - \frac{2^{j-1}d_{\max }^2}{2m}\omega ^2\lambda _0^2. \\ {}&\ge 2^{j-1} - \frac{2^{j-1}}{2} = 2^{j-2}. \end{aligned}$$
  The remainder of the proof is similar to the proof of Lemma 3.10 wherein Eq. (36) is replaced by
  $$\begin{aligned} 2^{j-2} \le \Delta _{p_r} \le \sum _{u \in V} d^+_{G_q}(u) \sum _{v \in N_0(u)}d^-_{G_q}(v). \end{aligned}$$
  This inequality can be shown to imply one of the following statements holds true:
  (i) $G_q$ has more than $2^{j/2-1}$ edges;
  (ii) for some $u \in V$, $\sum _{v \in N_0(u)} d^-_{G_q}(v) \ge 2^{j/2-1}$.
  Indeed, the probability that either of those statements holds, is upper bounded by $e^{-\Omega \left( 2^{j/2}\ln n \right) }$, using $\lambda _{j/2}=2^{j/2}(\ln n)^{1+\delta }$ in the same argument as in the proof of Lemma 3.10. Since *r* is arbitrary, this shows that $\mathbb {P}\left[ \Psi _r \ge 2^{j-1}\right] \le e^{-\Omega \left( 2^{j/2}\ln n\right) }$ for all *r* such that $m-r<\omega \lambda _0$, completing the proof.
- Since $B_j \subset S^*\left( \mathcal {M}\right)$ for all $1\le j \le K$, inequality (39) gives
  $$\begin{aligned} f\!\left( \mathcal {N}\right) \le e^{\sum _{r=0}^{m-1} \frac{4 \max \left( \Psi _r\left( \mathcal {N}\right) - \psi _r, 0\right) }{\tau (m-r)^2}}, \end{aligned}$$
  for all $\mathcal {N} \in B_j \setminus B_{j-1}$. According to the definition of $B_j$, we have
  $$\begin{aligned} \sum _{m-r=1}^{\omega \lambda _0} \frac{ \max \left( \Psi _r\left( \mathcal {N}\right) - \psi _r, 0\right) }{(m-r)^2} \le \sum _{m-r=1}^{\omega \lambda _0} \frac{2^j }{(m-r)^2} = \mathcal {O}\left( 2^j\right) , \end{aligned}$$
  and since $B_j \subset A_0$, the second statement from Lemma 3.14 can be applied, giving:
  $$\begin{aligned}\sum _{m-r= \omega \lambda _0}^{m} \frac{4 \max \left( \Psi _r\left( \mathcal {N}\right) - \psi _r, 0\right) }{\tau (m-r)^2} = o(1). \end{aligned}$$
  Hence for all $\mathcal {N} \in B_j$ it holds $f\!\left( \mathcal {N}\right) \le e^{\mathcal {O}\left( 2^j\right) + o(1)} = e^{\mathcal {O}\left( 2^j\right) }.$

$$\square $$

Now, we give a proof of asymptotic estimate (20). Lemma 3.15 implies that for all $B_j \setminus B_{j-1}$

$$\begin{aligned} \mathbb {E}\left[ f\!\left( \mathcal {N}\right) \mathbb {1}_{B_j\setminus B_{j-1}}\right] \le e^{-\Omega \left( 2^{j/2}\ln n\right) }e^{\mathcal {O}\left( 2^j\right) }. \end{aligned}$$

Recall that $j \le K$, and, in combination with Eq. (32), this yields $2^{\frac{j-1}{2}} \le \ln n$. Hence,

$$\begin{aligned} \mathbb {E}\left[ f\!\left( \mathcal {N}\right) \mathbb {1}_{\mathcal {B}}\right] = \sum _{j=1}^K\mathbb {E}\left[ f\!\left( \mathcal {N}\right) \mathbb {1}_{B_j\setminus B_{j-1}}\right] \le \sum _{j=1}^K e^{-\Omega \left( 2^{j/2}\ln n \right) }e^{\mathcal {O}\left( 2^j\right) } = o(1), \end{aligned}$$

proving Eq. (20).

**Proof of Equations** (21) **and** (22) We bound the expected value of $f\!\left( \mathcal {N}\right)$ for all $\mathcal {N} \in \mathcal {C}$. We start with proving upper bound (21), for which it suffices to show that for all $\mathcal {N} \in \mathcal {C}$,

$$\begin{aligned} f\!\left( \mathcal {N}\right) \le 1 + o(1). \end{aligned}$$

As $\mathcal {C} \subset S^*\left( \mathcal {M}\right)$, in analogy to Eq. (39), we have

$$\begin{aligned} f\!\left( \mathcal {N}\right)&= \prod _{r=0}^{m-1}\left( 1+\frac{\Psi _r\left( \mathcal {N}\right) - \psi _r}{(m-r)^2 - \Psi _r\left( \mathcal {N}\right) }\right) \\&\le \left( \prod _{m-r=1}^{ \lambda _0\omega } \left( 1+\frac{4 \max \left( \Psi _r\left( \mathcal {N}\right) - \psi _r, 0\right) }{\tau (m-r)^2} \right) \right) e^{\sum _{m-r=\lambda _0\omega +1}^{m} \frac{4 \max \left( \Psi _r\left( \mathcal {N}\right) - \psi _r, 0\right) }{\tau (m-r)^2}}. \end{aligned}$$

For the second term, since $\mathcal {C} \subset A_0$, we obtain from Lemma 3.14 that

$$\begin{aligned}\sum _{m-r=\lambda _0\omega +1}^{m} \frac{4 \max \left( \Psi _r\left( \mathcal {N}\right) - \psi _r, 0\right) }{\tau (m-r)^2}= o(1).\end{aligned}$$

For the first term, by definition of $\mathcal {C}$, we must have $\Psi _r \left( \mathcal {N}\right) \le 1$ for all $m-r \le \omega \lambda _0$. Furthermore, since $\Psi _r = \Delta _r + \Lambda _r$, $\Lambda _r>0$ and $\Delta _r\in {\mathbb {N}}$, we must have $\Delta _r=0$, and hence $\Psi _r =\Lambda _r \le \frac{d_{\max }^2}{2\,m}(m-r)^2$ by Lemma (3.2). Combining the latter bound with the bound on $\psi$ from Lemma (3.5) gives

$$\begin{aligned} \max \left( \Psi _r\left( \mathcal {N}\right) - \psi _r, 0\right) <(m-r)^2\mathcal {O}\left( \frac{d_{\max }^2}{m}\right) , \end{aligned}$$

Hence, for all $\mathcal {N} \in \mathcal {C}$,

$$\begin{aligned} f\!\left( \mathcal {N}\right)&\le \prod _{m-r=1}^{\lambda _0\omega }\left( 1 + \mathcal {O}\left( \frac{d_{\max }^2}{m}\right) \right) e^{o(1)} \le \left( 1 + \mathcal {O}\left( m^{-1/2}\right) \right) ^{\lambda _0\omega }e^{o(1)}=1 + o(1) \end{aligned}$$

proving Eq. (21).

Next, we derive a lower bound on $\mathbb {E}\left[ f\!\left( \mathcal {N}\right) \mathbb {1}_{S^*\left( \mathcal {M}\right) }\right]$. As $\mathcal {C} \subset S^*\left( \mathcal {M}\right)$, this will prove Eq. (22). Take any ordering $\mathcal {N} \in S^*\left( \mathcal {M}\right)$. Lemma 3.11 states that

41 $$\begin{aligned} \begin{aligned}&\mathbb {P}\left[ \left|\Psi _r\left( \mathcal {N}\right) - \psi _r \right|\ge 4 \beta _r\left( \lambda _0\right) + 2 \min \left( \gamma _r\left( \lambda _0\right) , \nu _r\right) \right] \le e^{-\Omega \left( \lambda _0\right) }\\&< e^{-( \ln n)^{1+\delta }} = o(1), \end{aligned} \end{aligned}$$

holds for all *r*, such that $m-r \ge \omega \lambda _0$. Thus, the probability that $\left|\Psi _r\left( \mathcal {N}\right) - \psi _r \right| \ge 4 \beta _r\left( \lambda _0\right) + 2 \min \left( \gamma _r\left( \lambda _0\right) , \nu _r\right)$ holds for at least one *r* is small. Now consider an ordering $\mathcal {N} \in S^*\left( \mathcal {M}\right)$ such that for all *r* with $m-r\ge \omega \lambda _0$,

42 $$\begin{aligned} \left|\Psi _r\left( \mathcal {N}\right) - \psi _r \right|\le 4 \beta _r\left( \lambda _0\right) + 2 \min \left( \gamma _r\left( \lambda _0\right) , \nu _r\right) . \end{aligned}$$

Recall that $\mathcal {N} \in S^*\left( \mathcal {M}\right)$ implies $\Psi _r\left( \mathcal {N}\right) \le \left( 1-\frac{\tau }{4}\right) (m-r)^2$. Combining this with the definition of $f\!\left( \mathcal {N}\right)$, we find:

$$\begin{aligned} f\!\left( \mathcal {N}\right)&\ge \prod _{m-r=\omega \lambda _0^3}^{m} \left( 1 - \frac{\Psi _r\left( \mathcal {N}\right) - \psi _r}{(m-r)^2 - \Psi _r\left( \mathcal {N}\right) }\right) \\&\quad \prod _{m-r=1}^{\omega \lambda _0^3 +1} \left( 1 - \frac{ \psi _r}{(m-r)^2 - \Psi _r\left( \mathcal {N}\right) }\right) \\&\ge \prod _{m-r = \omega \lambda _0^3 +1}^{m} \left( 1 - \frac{4}{\tau } \frac{4 \beta _r\left( \lambda _0\right) + 2 \min \left( \gamma _r\left( \lambda _0\right) , \nu _r\right) }{(m-r)^2 }\right) \\&\quad \prod _{m-r=1}^{\omega \lambda _0^3}\left( 1 -\frac{4}{\tau } \frac{\psi _r}{(m-r)^2 }\right) . \end{aligned}$$

From Lemma 3.14 and the definition of $T_r$, we find $\sum _{m-r= \omega \lambda _0^3 +1}^{m} \frac{4}{\tau }\frac{4 \beta _r\left( \lambda _0\right) + 2 \min \left( \gamma _r\left( \lambda _0\right) ,\nu _r\right) }{(m-r)^2} = o(1)$, which when combined with $1-x \ge e^{-2x}$ for $0 \le x\le \frac{1}{2}$, gives

$$\begin{aligned} f\!\left( \mathcal {N}\right)&\ge e^{-o(1)}\prod _{m-r=1}^{\omega \lambda _0^3}\left( 1 -\frac{4}{\tau } \frac{\psi _r}{(m-r)^2 }\right) . \end{aligned}$$

To approximate the remaining product, we apply Lemma 3.5 in combination with $1-x \ge e^{-2x}$ and an asymptotic estimate $\lambda _0^3\omega d_{\max }^2 = o(m)$ to obtain:

$$\begin{aligned} f\!\left( \mathcal {N}\right)&\ge e^{-2o(1)} \ge 1 - o(1). \end{aligned}$$

Now, for each $\mathcal {N} \in S^*\left( \mathcal {M}\right)$, we have shown that either $f\!\left( \mathcal {N}\right) \ge 1-o(1)$ or that its probability is upper bounded by *o*(1), which this completes the proof of Eq. (22). Remark that in fact we have proven

$$\begin{aligned} \mathbb {E}\left[ f\!\left( \mathcal {N}\right) \mathbb {1}_{S^*\left( \mathcal {M}\right) }\right] \ge 1 - o(1). \end{aligned}$$

Additionally, the proofs of Eqs. (19)–(22) demonstrate the following corollary.

#### Corollary 3.16

For a sufficiently large constant *c*, as used in the definition of $\lambda _L$,

$$\begin{aligned} \mathbb {E}\left[ \exp \left( \frac{1}{\tau ^2} \sum _{r=0}^{m-1} \frac{\max \left( \Psi _r\left( \mathcal {N}\right) - \psi _r, 0\right) }{(m-r)^2} \right) \right] = 1 + o(1). \end{aligned}$$

This corollary will be used to prove Eq. (23).

**Proving Eq.** (23). This equation is the last bit that remains to prove Eq. (6). It concerns the expected value of $f\!\left( \mathcal {N}\right)$ for the orderings in $S\left( \mathcal {M}\right) \setminus S^*\left( \mathcal {M}\right)$. Equation (15) implies that for any $\mathcal {N} \in S\left( \mathcal {M}\right) {\setminus } S^*\left( \mathcal {M}\right)$, there is at least one $0 \le r \le m-1$ that violates inequality

43 $$\begin{aligned} \Psi _r\left( \mathcal {N}\right) \le \left( 1-\frac{\tau }{4}\right) (m-r)^2. \end{aligned}$$

To deduce these values of *r*, let us assume that the above inequality is violated and investigate what are the implications for $\Delta _r$.

Recall that $\Psi _r = \Delta _r + \Lambda _r$. Using Lemma 3.2 to bound $\Lambda _r$, we obtain:

$$\begin{aligned} \Delta _r > \Psi _r - \frac{d_{\max }^2}{2m}(m-r)^2. \end{aligned}$$

Since $d_{\max }^4 = o(m)$, there is such $n_0$ that for all $n > n_0$, $\frac{d_{\max }^2}{m} < \frac{\tau }{2}$. Let $n > n_0$, then

$$\begin{aligned} \Delta _r&> \Psi _r - \frac{\tau }{4}(m-r)^2. \end{aligned}$$

Assuming the opposite inequality to (43) holds, this becomes:

44 $$\begin{aligned} \Delta _r&> \left( 1 - \frac{\tau }{2}\right) (m-r)^2. \end{aligned}$$

Lemma 3.2 states that $\Delta _r \le (m-r)d_{\max }^2$ and, hence, we deduce that $(m-r)\left( 1 - \frac{\tau }{2} \right) \le d_{\max }^2,$ which is equivalent to

$$\begin{aligned} m-r \le \frac{2d_{\max }^2}{2-\tau }. \end{aligned}$$

Therefore, inequality (43) can only be violated if $m-r \le \frac{2d_{\max }^2}{2-\tau }$.

We will now introduce partition

$$\begin{aligned} S\left( \mathcal {M}\right) \setminus S^*\left( \mathcal {M}\right) = \bigcup _{t =1}^{\frac{2d_{\max }^2}{2-\tau }} S_t\left( \mathcal {M}\right) , \end{aligned}$$

with $S_t\left( \mathcal {M}\right)$ being the set of all orderings $\mathcal {N}$ violating inequality (43) with $r = m-t$ and not violating it for all $r<m-t$. To prove Eq. (23), it suffices to show that

45 $$\begin{aligned} \mathbb {E}\left[ f\!\left( \mathcal {M}\right) \mathbb {1}_{S_t}\right] \le \mathcal {O}\left( \frac{1}{m^{t\tau }}\right) , \end{aligned}$$

for all $t \in \{1,2,\ldots , \frac{2d_{\max }^2}{2-\tau }\}$ as $\sum _{t=1}^\infty \frac{1}{m^{t\tau }} = o(1)$.

We will now prove Eq. (45). According to the definition of $\Psi _r,$ we have $(m-r)^2 - \Psi _r = \sum _{(u,v) \in E_r} {{d^+_{u}}}^{(r)}{{d^-_{ v}}}^{(r)} \left( 1-\frac{{d^+_{u}}{d^-_{ v}}}{2\,m}\right)$. For the algorithm to finish successfully, there must be at least $m-r$ suitable pairs left at each step *r*, implying that $(m-r)^2 - \Psi _r \ge (m-r)\left( 1-\frac{d_{\max }^2}{2\,m}\right)$. Therefore,

$$\begin{aligned} \frac{(m-r)^2}{(m-r)^2 - \Psi _r} \le \frac{(m-r)}{1 - \frac{d_{\max }^2}{2m}} = (m-r) \left( 1 + \mathcal {O}\left( \frac{d_{\max }^2}{2m}\right) \right) , \end{aligned}$$

and since $\frac{d_{\max }^4}{m}=o(1)$, we have: $\frac{(m-r)^2}{(m-r)^2 - \Psi _r} \le m-r+1$ for $m-r \le \frac{2d_{\max }^2}{2-\tau }$. Now we have that

$$\begin{aligned} \prod _{r=m-t}^{m-1} \frac{(m-r)^2- \psi _r}{(m-r)^2 - \Psi _r}&\le \prod _{r=m-t}^{m-1} \frac{(m-r)^2}{(m-r)^2 - \Psi _r} \\&\le \prod _{r=m-t}^{m-1} m-r+1 = (t+1)! \le t^t(t+1). \end{aligned}$$

In analogy to Eq. (39), it can also be shown that

$$\begin{aligned} \prod _{r=0}^{m-t} \frac{(m-r)^2 -\psi _r}{(m-r)^2 - \Psi _r} \le \exp \left[ \frac{4}{\tau } \sum _{r=0}^{m-1} \frac{\max (\Psi _r- \psi _r, 0)}{(m-r)^2}\right] . \end{aligned}$$

Combing these observations with inequality (43), which holds for all $r < m-t$, we find:

$$\begin{aligned} f\!\left( \mathcal {N}\right) \mathbb {1}_{S_t} \!=\!\mathbb {1}_{S_t}\prod _{r=0}^{m-r} \frac{(m-r)^2 \!-\!\psi _r}{(m-r)^2 \!-\! \Psi _r}\!\le \! \mathbb {1}_{S_t} \exp \left[ \frac{4}{\tau } \!\sum _{r=0}^{m-1} \frac{\max (\Psi _r\!-\! \psi _r, 0)}{(m-r)^2}\right] t^t(t+1). \end{aligned}$$

Next, we take the expected value of the above equation and apply Hölder’s inequality to obtain:

$$\begin{aligned} \mathbb {E}\left[ f\!\left( \mathcal {N}\right) \mathbb {1}_{S_t}\right] \le \mathbb {E}\left[ \mathbb {1}_{S_t}\right] ^{1-\tau } \mathbb {E}\left[ \mathbb {1}_{S_t} \exp \left[ \frac{4}{\tau ^2} \sum _{r=0}^{m-1} \frac{\max (\Psi _r- \psi _r, 0)}{(m-r)^2}\right] \right] ^{\tau } t^t(t+1). \end{aligned}$$

Using Corollary 3.16, this becomes

$$\begin{aligned} \mathbb {E}\left[ f\!\left( \mathcal {N}\right) \mathbb {1}_{S_t}\right] \le \mathbb {E}\left[ \mathbb {1}_{S_t}\right] ^{1-\tau } \left[ 1+o(1)\right] t^t(t+1). \end{aligned}$$

Hence, to prove Eq. (45), it remains to show that

46 $$\begin{aligned} \mathbb {P}\left[ \mathcal {N} \in S_t\right] ^{1-\tau } t^t(t+1) \le \left[ 1+o(1)\right] \frac{1}{{m^{\tau t}}}. \end{aligned}$$

We will now bound $\mathbb {P}\left[ \mathcal {N} \in S_t\right]$ from above using the following rationale: As the first step, we show that if $\mathcal {N} \in S_t$, then $G_{\mathcal {N}_r}$ always contains a vertex with some special property. We use the probability that such a vertex exists as an upper bound for $\mathbb {P}\left[ \mathcal {N} \in S_t\right]$. Let us assume that $\mathcal {N} \in S_t$, fix $r = m-t$ and define $\Gamma (u):= \{ v \in V| (u,v) \in G_{\mathcal {N}_r}\}$. By definition of $\Delta _r$, this allows us to write

$$\begin{aligned} \Delta _r =\sum _{u \in V} {{d^+_{u}}}^{(r)} \sum _{v \in \Gamma (u) \cup \{u\}} {{d^-_{ v}}}^{(r)}\quad \text {and}\quad (m-r)^2 = \sum _{u \in V} {{d^+_{u}}}^{(r)} \sum _{v \in V} {{d^-_{ v}}}^{(r)}. \end{aligned}$$

Because $\mathcal {N} \in S_t$, inequality (44) must hold. Inserting the above expressions for $\Delta _r$ and $(m-r)$ into this inequality yields:

$$\begin{aligned} \sum _{u \in V} {{d^+_{u}}}^{(r)} \sum _{v \in \Gamma (u) \cup \{u\}} {{d^-_{ v}}}^{(r)}&> \left( 1 - \frac{\tau }{2}\right) \sum _{u \in V} {{d^+_{u}}}^{(r)} \sum _{v \in V} {{d^-_{ v}}}^{(r)} \\&> \left( 1 - \tau \right) \sum _{u \in V} {{d^+_{u}}}^{(r)} \sum _{v \in V} {{d^-_{ v}}}^{(r)}, \end{aligned}$$

which implies that there exists a vertex $u \in V$ such that

47 $$\begin{aligned} {{d^+_{u}}}^{(r)}> 0 \quad \text {and} \quad \sum _{ v \in \Gamma (u) \cup \{u\}} {{d^-_{ v}}}^{(r)} > (1-\tau ) \sum _{v \in V} {{d^-_{ v}}}^{(r)} = (1-\tau )t. \end{aligned}$$

Thus, we have shown that if $\mathcal {N} \in S_t$, there must exist a vertex *u* obeying (47), and therefore, probability that $G_{\mathcal {N}_r}$ contains such a vertex *u* provides an upper bound for $\mathbb {P}\left[ \mathcal {N} \in S_t\right]$.

Next, we derive an upper bound on the probability that *u* obeys (47). Recall that $G_{\mathcal {N}_r}$ contains the first *r* edges of the ordering $\mathcal {N}$. Adding the remaining *t* edges of $\mathcal {N}$ completes $G_{\textbf{d}}$. In this complement edge set, let *l* out of *t* edges have their target in $\Gamma (u) \cup \{u\}$, then $l = \sum _{ v \in \Gamma (u) \cup \{u\}} {{d^-_{ v}}}^{(r)}$. Let $k:= {d^+_{u}} - \left|\Gamma (u) \right|={{d^+_{u}}}^{(r)}$. Inequality (47) holds if and only if $k \ge 1$ and $l \ge (1-\tau )t$. We derive an upper bound on the probability that $k \ge 1$ and $l \ge (1-\tau )t$ for a random ordering $\mathcal {N} \in S\left( \mathcal {M}\right)$. That is to say we fix all *m* edges in the graph, but the order in which they are drawn $\mathcal {N}$ is a uniform random variable. To obtain a fixed value of *k*, exactly *k* of the ${d^+_{u}}$ edges with *u* as source must be in $\mathcal {N}{\setminus } \mathcal {N}_r$. Choosing these edges determines $\Gamma (u)$. To obtain the desired value of *l*, exactly *l* edges with target in $\Gamma (u) \cup \{u\}$ must be in $\mathcal {N}{\setminus } \mathcal {N}_r$. There are $\sum _{v \in \Gamma (u) \cup \{u\}} \left( {d^-_{ v}}-1\right) + {d^-_{ u}}$ edges to choose from, since for each $v \in \Gamma (u)$, the edge with *v* as the target and *u* as the source is already in $\mathcal {N}_r$. The remaining $t-l-k$ edges that are not in $\mathcal {N}_r$ may be chosen freely amongst all the edges that do not have *u* as a source or an element of $\Gamma (u) \cup \{u\}$ as target. Thus, the probability to get a specific combination of *k* and *l* is

$$\begin{aligned} \frac{ {{d^+_{u}} \atopwithdelims ()k}{{\sum _{v \in \Gamma (u)} \left( {d^-_{ v}}-1\right) + {d^-_{ u}}} \atopwithdelims ()l}{{m-{d^+_{u}} -\sum _{v \in \Gamma (u) \cup } \left( {d^-_{ v}}-1\right) - {d^-_{ u}} }\atopwithdelims (){t-l-k}}}{{m \atopwithdelims ()t}}. \end{aligned}$$

We, therefore, write the upper bound for the probability that a randomly chosen vertex *u* satisfies (47) as

$$\begin{aligned} \sum _{k\ge 1, l \ge (1-\tau )t} \frac{{{d^+_{u}} \atopwithdelims ()k}{{({d^+_{u}}-k+1)d_{\max }} \atopwithdelims ()l}{{m-{d^+_{u}} -\sum _{v \in \Gamma (u)} \left( {d^-_{ v}}-1\right) - {d^-_{ u}}}\atopwithdelims (){t-l-k}}}{{m\atopwithdelims ()t}}. \end{aligned}$$

For $\mathcal {N} \in S_t$, at least one vertex satisfies inequality (47); thus, using the union bound gives:

$$\begin{aligned} \mathbb {P}\left[ \mathcal {N} \in S_t\right] \le \sum _{u \in V} \sum _{k\ge 1, l \ge (1-\tau )t} \frac{{{d^+_{u}} \atopwithdelims ()k}{{({d^+_{u}}-k+1)d_{\max }} \atopwithdelims ()l}{{m-{d^+_{u}} -\sum _{v \in \Gamma (u)} \left( {d^-_{ v}}-1\right) - {d^-_{ u}}}\atopwithdelims (){t-l-k}}}{{m\atopwithdelims ()t}}. \end{aligned}$$

Remark that ${m \atopwithdelims ()t} \le \frac{m^t}{t!}$, and since $t = \mathcal {O}\left( d_{\max }^2\right)$ and $\mathcal {O}\left( d_{\max }^4\right) = o(m)$,

$$\begin{aligned} {m\atopwithdelims ()t} = \left[ 1 + o(1)\right] \frac{m^t}{t!}. \end{aligned}$$

This gives

$$\begin{aligned} \mathbb {P}\left[ S_t\right]&\le \sum _{u \in V}\sum _{k \ge 1, l \ge (1-\tau )t} \left[ 1 + o(1)\right] \frac{{{d^+_{u}}}^k \left( ({d^+_{u}}-k+1)(d_{\max })\right) ^l m^{t-l-k}t!}{m^t k! l! (m-l-k)!}\\&= \sum _{u \in V}\sum _{k \ge 1, l \ge (1-\tau )t} \left[ 1 + o(1)\right] \frac{\left( \frac{{d^+_{u}}}{m}\right) ^k \left( \frac{({d^+_{u}}-k+1)d_{\max }}{m}\right) ^l t!}{k! l! (m-l-k)!}. \end{aligned}$$

Finally, we approximate the sum over *k* and *l*. Since adding *t* edges completes the ordering, $\sum _{u \in V}{{d^+_{u}}}^{(r)} = \sum _{u \in V} {{d^-_{ u}}}^{(r)} = t$. This implies that $k \in \{1,2,\ldots t\}$ and that *l* is an integer in the interval $[(1-\tau )t, t]$. Thus, this sum consists of at most $t\tau$ terms. Remark that, as $l,k \le t = \mathcal {O}\left( d_{\max }^2\right) = \mathcal {O}\left( m^{1/2}\right) , \left( \frac{{d^+_{u}}}{m}\right) = \mathcal {O}\left( \frac{1}{m^{3/4}}\right)$ and $\left( ({d^+_{u}}-k+1)(d_{\max })\right) = \mathcal {O}\left( \frac{1}{m^{1/2}}\right)$, the term inside the summation is maximal for $k=1$ and $l = \left( 1-\tau \right) t$. This gives:

$$\begin{aligned} \mathbb {P}\left[ S_t\right]&\le \left[ 1 + o(1)\right] \tau t \sum _{u \in V} \left( \frac{{d^+_{u}}}{m}\right) \left( \frac{{d^+_{u}} d_{\max } }{m}\right) ^{(1-\tau )t} {t\atopwithdelims (){t\tau }}\\&\le \left[ 1 + o(1)\right] 2^t t \left( \frac{d_{\max }^2 }{m}\right) ^{(1-\tau )t} \sum _{v \in V} \left( \frac{{d^+_{u}}}{m}\right) \\&\le \left[ 1 + o(1)\right] 2^t t \left( \frac{ d_{\max }^2 }{m}\right) ^{(1-\tau )t}. \end{aligned}$$

Here we used that $\tau \le \frac{1}{3}, {m\atopwithdelims ()k}\le 2^m$ and $\sum _{u \in V} {d^+_{u}} = m$. Plugging this into (46) yields:

$$\begin{aligned} \mathbb {P}\left[ \mathcal {N} \in {S_t}\right] ^{1-\tau }t^t (t+1)&\le \left[ 1 + o(1)\right] t^t(t+1) \left( 2^t t \left( \frac{ d_{\max }^2 }{m}\right) ^{(1-\tau )t}\right) ^{1-\tau }. \end{aligned}$$

Since $t \le \frac{2d_{\max }^2}{2-\tau }$, we have:

$$\begin{aligned} \mathbb {P}\left[ \mathcal {N} \in {S_t}\right] ^{1-\tau }t^t (t+1)&\le \left[ 1 + o(1)\right] (t+1) t ^{1-\tau } \left( \frac{2\cdot 2^{1-\tau }}{2-\tau }\frac{ d_{\max }^{4 - 4\tau + 2\tau ^2} }{m^{1 - 2\tau + \tau ^2}}\right) ^{t}, \end{aligned}$$

and since $\tau \le \frac{1}{3}$, for any $x \ge 1$, $x^{1-\tau } \le x$, we find:

$$\begin{aligned} \mathbb {P}\left[ \mathcal {N} \in {S_t}\right] ^{1-\tau }t^t (t+1)&\le \left[ 1 + o(1)\right] (t+1) t \left( \frac{4}{2-\tau }\frac{ d_{\max }^{4 - 4\tau + 2\tau ^2} }{m^{1 - 2\tau + \tau ^2}}\right) ^{t}. \end{aligned}$$

Inserting the estimate $d_{\max } = \mathcal {O}\left( m ^{1/4-\tau }\right)$ yields,

$$\begin{aligned} \mathbb {P}\left[ \mathcal {N} \in {S_t}\right] ^{1-\tau }t^t (t+1)&\le \left[ 1 + o(1)\right] (t+1) t \left( \frac{4}{2-\tau }m^{-3\tau + 3.5\tau ^2 - 3\tau ^3}\right) ^{t}, \end{aligned}$$

and using $t = o\left( m^{1/2}\right)$ and that $\frac{4}{2-\tau }$ is constant when *m* goes to infinity with *n*, we find:

$$\begin{aligned} \mathbb {P}\left[ \mathcal {N} \in {S_t}\right] ^{1-\tau }t^t (t+1)&\le \left[ 1+o(1)\right] o\left( m^{1/2}\right) \mathcal {O}\left( m^{-3\tau + 3.5\tau ^2 - 3\tau ^3}\right) ^{t} = \mathcal {O}\left( m^{-\tau t}\right) . \end{aligned}$$

This completes the proof of inequality (45), and hence it shows that Eq. (23) holds. This completes the prove of Eq. (13), and hence proves Eq. (5). Together with the results from the beginning of Sect. 3 and Sect. 3.2, this completes the proof of Theorem 2.2.

## The Probability of Failure of Algorithm 1

Here we show that the probability the algorithm fails is *o*(1). The proof is inspired by Ref. [3, Section 5]. If at step *s*, every pair of an unmatched in-stub with an unmatched out-stub is unsuitable—the algorithm fails. In this case, the algorithm will necessarily create a self-loop or double edge when the corresponding edge is added to $G_{\mathcal {N}_s}$. First, we investigate at which steps $s \in \{0,1,\ldots , m-1\}$ the algorithm can fail. Then we derive an upper bound for the number of vertices that are left with unmatched stubs when the algorithm fails. For a given number of unmatched stubs, this allows us to determine the probability that the algorithm fails. Combining these results, we show that this probability is *o*(1). The following lemma states that the algorithm has to be close to the end to be able to fail.

### Lemma 4.1

If Algorithm 1 fails at step *s*, then $m-s \le d_{\max }^2$.

### Proof

At step *s*, there are $(m-s)^2$ pairs of unmatched stubs. If the algorithm fails at step *s*, all these pairs are unsuitable. The number of unsuitable pairs at step *s* is $\Delta _s$. According to Lemma 3.2, $\Delta _s \le d_{\max }^2(m-s)$. Therefore, if the algorithm fails at step *s*, $(m-s)^2 \le d_{\max }^2(m-s)$. $\square$

The number of vertices that have unmatched stubs when the algorithm fails is also bounded. Suppose a vertex $v \in V$ has unmatched in-stub(s) left when the algorithm fails. Since the number of unmatched in-stubs equals the number of unmatched out-stubs, this implies that there are also unmatched out-stubs. Because the algorithm fails, every pair of an unmatched in-stub and an unmatched out-stub induces either a double edge or self-loop. Hence, only *v* and vertices that are the source of an edge with *v* as a target can have unmatched out-stub(s). As *v* has at least one unmatched in-stub, there are atmost $d_{\max }-1$ edges with *v* as a target. Thus, atmost $d_{\max }$ vertices have unmatched out-stub(s). Symmetry implies that atmost $d_{\max }$ vertices have unmatched in-stub(s) when a failure occurs.

Let $A_{{d_{i_1}^-}^{(s)}, \ldots , {d_{i_{k^-}}^-}^{(s)}, {d_{j_1}^+}^{(s)}, \ldots {d_{j_{k^+}}^+}^{(s)}}$ be the event that the algorithm fails at step *s* with $v_{i_1}, \ldots , v_{i_{k^-}}\in V$ being the only vertices with unmatched in-stubs and $v_{j_1}, \ldots , v_{j_{k^+}}$ the only vertices having unmatched out-stubs. The amount of unmatched in-stubs (respectively out-stubs) of such a vertex $i_l$ ($j_l$) is denoted by ${d_{i_l}^-}^{(s)}$ ( ${d_{j_l}^-}^{(s)}$). Since $k^-$ (respectively $k^+$) denotes the number of vertices with unmatched in-stubs (out-stubs) that are left, $k^-,k^+ \le d_{\max }.$ This allows to write the probability that Algorithm 1 fails as

48 $$\begin{aligned} \mathbb {P}\left[ \,\text {failure}\,\right]&= \sum _{m-s = 1}^{d_{\max }^2} \sum _{k^-,k^+ = 1}^{\max \left( m-s, d_{\max }\right) } \sum _{i_1, \ldots , i_{k^-} = 1}^{n} \sum _{j_1,\ldots , j_{k^+} =1}^n\nonumber \\&\quad \quad \mathbb {P}\left[ A_{{d_{i_1}^-}^{(s)}, \ldots , {d_{i_{k^-}}^-}^{(s)}, {d_{j_1}^+}^{(s)}, \ldots {d_{j_{k^+}}^+}^{(s)}}\right] . \end{aligned}$$

The sum $\sum _{i_1, \ldots , i_{k^-} = 1}^{n}$ is the sum over all possible subsets $B \subset \{1,2,\ldots , n\}$ of size $k^-$, such that $\sum _{i \in B} {d_{i}^-}^{(s)} = m-s$ and $\sum _{ i \notin B} {d_{i}^-}^{(s)} = 0$. The goal is to show that $\mathbb {P}\left[ \,\text {failure}\,\right] = o(1)$, which we achieve by first determining an upper bound for $\mathbb {P}\left[ A_{{d_{i_1}^-}^{(s)}, \ldots , {d_{i_{k^-}}^-}^{(s)}, {d_{j_1}^+}^{(s)}, \ldots {d_{j_{k^+}}^+}^{(s)}}\right]$.

### Lemma 4.2

The probability of the event $A_{{d_{i_1}^-}^{(s)}, \ldots , {d_{i_{k^-}}^-}^{(s)}, {d_{j_1}^+}^{(s)}, \ldots {d_{j_{k^+}}^+}^{(s)}}$ is upper bounded by

49 $$\begin{aligned}&e^{o(1)}d_{\max }^{2k^+k^- - 2k^\pm } \frac{\prod _{i \in K^+} {{d^+_{i}}}^{ {{d^+_{i}}}^{(s)}}\prod _{i \in K^-} {{d^-_{ i}}}^{ {{d^-_{ i}}}^{(s)}}}{m^{k^{+}k^{-} - k^{\pm }} m^{2(m-s)}}\nonumber \\&\quad {{m-s}\atopwithdelims (){{d_{i_1}^-}^{(s)}, \ldots ,{d_{i_{k^-}}^-}^{(s)} } }{{m-s} \atopwithdelims (){{d_{j_1}^+}^{(s)}, \ldots ,{d_{j_{k^+}}^+}^{(s)} } }. \end{aligned}$$

### Proof

Let us define $K^- := \left\{ i_1,i_2,\ldots i_{k^-} \right\} , \; K^+ := \left\{ j_1, j_2, \ldots j_{k^+}\right\} , \text { and } K^{\pm } := K^- \cap K^+.$ When event $A_{{d_{i_1}^-}^{(s)}, \ldots , {d_{i_{k^-}}^-}^{(s)}, {d_{j_1}^+}^{(s)}, \ldots {d_{j_{k^+}}^+}^{(s)}}$ occurs, the algorithm has constructed a graph $G_{\mathcal {M}_s}$ having the degree sequence ${\widetilde{\textbf{d}}}$ with elements:

$$\begin{aligned} \widetilde{{d^-_{ i}}} = {\left\{ \begin{array}{ll} {d^-_{ i}} &{} \text {if} \; i \notin K^- \\ {d^-_{ i}} - {{d^-_{ i}}}^{(s)} &{} \text {if} \; i \in K^- \\ \end{array}\right. }, \quad \widetilde{{d^-_{ i}}} = {\left\{ \begin{array}{ll} {d^+_{i}} &{} \text {if} \; i \notin K^+ \\ {d^+_{i}} - {{d^+_{i}}}^{(s)} &{} \text {if} \; i \in K^+ \\ \end{array}\right. }. \end{aligned}$$

The probability of $A_{{d_{i_1}^-}^{(s)}, \ldots , {d_{i_{k^-}}^-}^{(s)}, {d_{j_1}^+}^{(s)}, \ldots {d_{j_{k^+}}^+}^{(s)}}$ equals the number of graphs $G_{\mathcal {M}_s}$ that obey ${\widetilde{\textbf{d}}}$ and lead to a failure multiplied by the probability that the algorithm constructs this partial graph. To construct an upper bound on the number of graphs obeying ${\widetilde{\textbf{d}}}$ and leading to a failure, note that such a graph must contain the edge (*i*, *j*) for all $i \in K^+, j \in K^-, i \ne j$ and, therefore, it must contain a subgraph obeying degree sequence $\overline{d_{K^-,K^+}}^{(s)}$, which is defined by:

$$\begin{aligned} \overline{d^{-}_i}^{(s)}:= {\left\{ \begin{array}{ll} {d^-_{ i}} &{} \text {if}\; i \notin K^-\\ {d^-_{ i}} - {d_1^-}^{(s)} - k^+ &{} \text {if}\; i \in K^-, i \notin K^+\\ {d^-_{ i}} - {d_1^-}^{(s)} - k^+ +1 &{} \text {if}\; i \in K^-, i \in K^+\\ \end{array}\right. } \end{aligned}$$

and

$$\begin{aligned} \overline{{d^+_{i}}}^{(s)} := {\left\{ \begin{array}{ll} {d^+_{i}} &{} \text {if}\; i \notin K^+\\ {d^+_{i}} - {d_1^+}^{(s)} - k^- &{} \text {if}\; i \in K^+, i \notin K^-\\ {d^+_{i}} - {d_1^+}^{(s)} - k^-+1 &{} \text {if}\; i \in K^+, i \in K^-\\ \end{array}\right. }. \end{aligned}$$

The number of graphs obeying the degree sequence $\overline{d_{K^-,K^+}}^{(s)}$ gives an upper bound for the number of partial graphs inducing event $A_{{d_{i_1}^-}^{(s)}, \ldots , {d_{i_{k^-}}^-}^{(s)}, {d_{j_1}^+}^{(s)}, \ldots {d_{j_{k^+}}^+}^{(s)}}$. Denote by $\mathcal {L}\left( \textbf{d}\right)$ the space of simple graphs obeying the degree sequence $\textbf{d}$. Theorem 2.2 implies that for any degree sequence *d* with $d_{\max } = \mathcal {O}\left( m^{1/4-\tau }\right)$,

50 $$\begin{aligned}&\left|\mathcal {L}\left( d\right) \right|\le \frac{\prod _{r=0}^{m-1} (m-r)^2}{m! \prod _{i=1}^n {d^+_{i}}! \prod _{i=1}^n {d^-_{ i}}!}\nonumber \\&\quad \quad e^{-\frac{\sum _{i=1}^n {d^-_{ i}}{d^+_{i}}}{m} + \frac{\sum _{i=1}^n ({d^-_{ i}})^2 + ({d^+_{i}})^2}{2m} - \frac{\sum _{i=1}^n({d^-_{ i}})^2\sum _{i=1}^n({d^+_{i}})^2}{2m^2} -\frac{1}{2} + o(1)}. \end{aligned}$$

We apply this bound to the degree sequence $$\overline{d_{K^-,K^+}}^{(s)}$$. A graph obeying this degree sequence has $$s-k^+k^-+k^{\pm }$$ edges, with $$k^\pm = |K^\pm |$$. Thus, we must show that $$d_{\max } = \mathcal {O}\left( \left( s-k^-k^++k^\pm \right) ^{1/4-\tau }\right) $$. Combining the statement of Lemma 4.1 with $$d_{\max }^4 = o(m)$$ gives $$ s > 3d_{max}^2$$ for $$d_{\max }>1$$. Since $$k^+k^- \le d_{\max }^2$$, we now find $$m < 2\left( s - k^-k^+ + k^\pm \right) $$, that is $$m = \mathcal {O}\left( s - k^-k^+ + k^\pm \right) $$, which implies that $$ d_{\max } = \mathcal {O}\left( m^{1/4-\tau }\right) = \mathcal {O}\left( \left( s - k^-k^+ k^\pm \right) ^{1/4-\tau }\right) .$$ Thus, we may apply inequality (50) to $$\overline{d_{K^-,K^+}}^{(s)}$$ to obtain

$$\begin{aligned}&\left|\mathcal {L}\left( \overline{d_{K^-,K^+}}^{(s)}\right) \right|\le \frac{\left( s-k^+k^- + k^\pm \right) !}{\prod _{i=1}^n \overline{{d^+_{i}}}^{(s)}! \prod _{i=1}^n \overline{{d^-_{ i}}}^{(s)}!} \\ {}&\quad \times \exp \!\left( \frac{\sum _{i=1}^n \left[ (\overline{{d^-_{ i}}}^{(s)})^2 + (\overline{{d^+_{i}}}^{(s)})^2\right] }{2\left( s-k^+k^-+k^{\pm }\right) }\right. \\&\quad \left. -\frac{\sum _{i=1}^n \overline{{d^-_{ i}}}^{(s)}\overline{{d^+_{i}}}^{(s)}}{s-k^+k^-+k^{\pm }} - \frac{\sum _{i=1}^n(\overline{{d^-_{ i}}}^{(s)})^2\sum _{i=1}^n(\overline{{d^+_{i}}}^{(s)})^2}{2\left( s-k^+k^-+k^{\pm }\right) ^2} -\frac{1}{2} + o(1)\right) . \end{aligned}$$

Following the derivation in Sect. 3, we find

$$\begin{aligned} \mathbb {P}\left( G_{\mathcal {M}_s}\right)&=\frac{\prod _{i=1}^n {d^+_{i}}!\prod _{i=1}^n {d^-_{ i}}!}{\prod _{i \in K^+} {{d^+_{i}}}^{(s)}! \prod _{i \in K^-} {{d^-_{ i}}}^{(s)}!}\sum _{\mathcal {N}_s \in S\left( \mathcal {M}_s\right) } \mathbb {P}\left( \mathcal {N}_s\right) \\&=\frac{\prod _{i=1}^n {d^+_{i}}!\prod _{i=1}^n {d^-_{ i}}!}{\prod _{i \in K^+} {{d^+_{i}}}^{(s)}! \prod _{i \in K^-} {{d^-_{ i}}}^{(s)}!}s!\prod _{r=0}^{s-1}\frac{1}{(m-r)^2} \\&\qquad \times \exp \left( \frac{s\sum _{i=1}^n {d^-_{ i}}{d^+_{i}}}{m^2} - \frac{s^2\sum _{i=1}^n \left[ ({d^-_{ i}})^2 +({d^+_{i}})^2\right] }{2m^3}\right. \\&\qquad \left. + \frac{s\sum _{i=1}^n ({d^-_{ i}})^2 \sum _{i=1}^n({d^+_{i}})^2}{2m^3} + \frac{s^2}{2m^2} + o(1) \right) . \end{aligned}$$

In the latter expression, the factor with factorials accounts for the number of different configurations leading to the same graph $G_{\mathcal {M}_s}$, which equals the number of permutations of the stub labels. However, for $i \in K^{-}$, there are only $\frac{{d^-_{ i}}!}{{{d^-_{ i}}}^{(s)}!}$ permutations of the labels of the in-stubs of $v_i$ that lead to a different configuration. Remark that changing the label of an in-stub that remains unmatched with another in-stub that remains unmatched does not change the configuration. By the same argument for $i \in K^{+}$, there are only $\frac{{d^+_{i}}!}{{{d^+_{i}}}^{(s)}!}$ ways to permute the labels of the out-stubs of $v_i$.

We can now determine

$$\begin{aligned}&\mathbb {P}\left[ A_{{d_{i_1}^-}^{(s)}, \ldots , {d_{i_{k^-}}^-}^{(s)}, {d_{j_1}^+}^{(s)}, \ldots {d_{j_{k^+}}^+}^{(s)}}\right] \le \mathbb {P}\left[ G_{\mathcal {M}_s}\right] \left|\mathcal {L}\left( {\bar{d}}_{k^-,k^+}^{(s)}\right) \right|. \end{aligned}$$

First, we look at the product of the exponentials in the asymptotic approximations of $\mathbb {P}\left[ G_{\mathcal {M}_s}\right]$ and $\left|\mathcal {L}\left( {\bar{d}}_{k^-,k^+}^{(s)}\right) \right|$, which after some transformations, and using that $m > s \ge m - d_{\max }^2$, becomes:

$$\begin{aligned}&\exp \left( \frac{\sum _{i=1}^n \left[ (\overline{{d^-_{ i}}}^{(s)})^2 + (\overline{{d^+_{i}}}^{(s)})^2\right] }{2\left( s-k^+k^-+k^{\pm }\right) } -\frac{\sum _{i=1}^n \overline{{d^-_{ i}}}^{(s)}\overline{{d^+_{i}}}^{(s)}}{s-k^+k^-+k^{\pm }} \right. \\&\quad \left. - \frac{\sum _{i=1}^n(\overline{{d^-_{ i}}}^{(s)})^2\sum _{i=1}^n(\overline{{d^+_{i}}}^{(s)})^2}{2\left( s-k^+k^-+k^{\pm }\right) ^2} - \frac{1}{2} + o(1)\right) \\&= \exp \left( \frac{s}{m}\mathcal {O}\left( d_{\max }\right) + \frac{s}{m} \mathcal {O}\left( d_{\max }^2\right) +o(1)\right) \\&\quad \exp \left( -\mathcal {O}\left( d_{\max }\right) - \mathcal {O}\left( d_{\max }^2\right) +o(1)\right) = e^{o(1)}. \end{aligned}$$

Using the latter estimate, we obtain

$$\begin{aligned}&\mathbb {P}\left[ A_{{d_{i_1}^-}^{(s)}, \ldots , {d_{i_{k^-}}^-}^{(s)}, {d_{j_1}^+}^{(s)}, \ldots {d_{j_{k^+}}^+}^{(s)}}\right] \le \mathbb {P}\left[ G_{\mathcal {M}_s}\right] \left|\mathcal {L}\left( \overline{d_{K^-,K^+}}^{(s)}\right) \right|\\&\quad \le e^{o(1)} \frac{\prod _{i \in K^+} {d^+_{i}}!\prod _{i \in K^-} {d^-_{ i}}! \prod _{i \in K^+, i \in K^-}\left( {d^+_{i}} - {{d^+_{i}}}^{(s)} -k^- \right) \left( {d^-_{ i}} - {{d^-_{ i}}}^{(s)} - k^+\right) }{\prod _{i \in K^+} \left( {d^+_{i}} - {{d^+_{i}}}^{(s)} - k^-\right) !{{d^+_{i}}}^{(s)}! \prod _{i \in K^-} \left( {d^-_{ i}} - {{d^-_{ i}}}^{(s)} - k^+\right) !{{d^-_{ i}}}^{(s)}!} \\&\qquad \times \frac{(s- k^+k^- + k^\pm )!s! (m-s)! (m-s)!}{m! m!}\\&\quad \le e^{o(1)}d_{\max }^{2k^+k^- - 2k^\pm }\prod _{i \in K^+} {{d^+_{i}}}^{ {{d^+_{i}}}^{(s)}}\prod _{i \in K^-} {{d^-_{ i}}}^{ {{d^-_{ i}}}^{(s)}} \frac{1}{\prod _{j=0}^{k^+k^- - k^\pm +1} s-j} \left( \frac{s! }{m!}\right) ^2\\&\qquad \times {{m-s} \atopwithdelims (){{d_{i_1}^-}^{(s)}, \ldots ,{d_{i_{k^-}}^-}^{(s)} } }{{m-s} \atopwithdelims (){{d_{j_1}^+}^{(s)}, \ldots ,{d_{j_{k^+}}^+}^{(s)} } } . \end{aligned}$$

It remains to bound $\frac{s!}{m!}$ and $\frac{\prod _{j=0}^{k^+k^- - k^\pm +1} s-j }{m^{k^+k^- - k^\pm }}$. First, using that $m-s = \mathcal {O}\left( d_{\max }^2\right)$, we find:

$$\begin{aligned} \frac{m!}{s!}&= (s+1)(s+2) \cdots (m-1)m \\&= m^{m-s}\left( 1 -\frac{1}{m}\right) \left( 1-\frac{2}{m}\right) \cdots \left( 1-\frac{m-s-1}{m}\right) \\&= m^{m-s} \left( 1 - \prod _{i=1}^{m-s-1}\frac{i}{m} + \mathcal {O}\left( (m-s)^2\frac{(m-s)^2}{m^2}\right) \right) \\&\ge m^{m-s}e^{-\sum _{i=1}^{m-s-1}\frac{i}{m} + \mathcal {O}\left( \frac{d_{\max }^8}{m^2}\right) } \\&= m^{m-s}e^{-\frac{(m-s)(m-s-1)}{2m} + \mathcal {O}\left( \frac{d_{\max }^8}{m^2}\right) }= m^{m-s}e^{-\mathcal {O}\left( \frac{d_{\max }^4}{m}\right) }, \end{aligned}$$

and therefore $\frac{s!}{m!} \le \frac{1}{m^{m-s}}e^{\mathcal {O}\left( \frac{d_{\max }^4}{m}\right) } = \frac{1}{m^{m-s}} e^{o(1)}.$ Second, let us consider $\frac{1}{\prod _{j=0}^{k^+k^- - k^\pm +1} s-j }$. Using that $m-s \le d_{\max }^2, k^+,k^- \le d_{\max }$ and $0 \le k^\pm \le \min \left( k^-,k^+\right)$, we obtain:

$$\begin{aligned} \prod _{j=0}^{k^+k^- - k^\pm +1} s-j&\ge \prod _{j=0}^{k^+k^- - k^\pm +1} m -d_{\max }^2-j\\&= m^{k^+k^- - k^{\pm }}\prod _{j=0}^{k^+k^- - k^\pm +1} \left( 1- \frac{d_{\max }^2+j}{m} \right) \\&= m^{k^+k^- -k^\pm } \left( 1 - \prod _{j=1}^{k^+k^- - k^\pm +1}\frac{d_{\max }^2 + j}{m} + \mathcal {O}\left( \frac{d_{\max }^8}{m^2}\right) \right) \\&\ge m^{k^+k^- - k^\pm }e^{-\frac{(d_{\max }^2 + k^+k^- +k^\pm +1)(d_{\max }^2 + k^+k^- +k^\pm +2)}{2m} + \mathcal {O}\left( \frac{d_{\max }^8}{m^2}\right) }\\&= m^{k^+k^- - k^\pm }e^{-\mathcal {O}\left( \frac{d_{\max }^4}{m}\right) } , \end{aligned}$$

which gives

$$\begin{aligned} \frac{1}{\prod _{j=0}^{k^+k^- - k^\pm +1} s-j } \le \frac{1}{m^{k^+k^- - k^\pm }}e^{\mathcal {O}\left( \frac{d_{\max }^4}{m}\right) } = \frac{1}{m^{k^+k^- - k^\pm }} e^{o(1)}. \end{aligned}$$

Thus the upper bound on the probability of $A_{{d_{i_1}^-}^{(s)}, \ldots , {d_{i_{k^-}}^-}^{(s)}, {d_{j_1}^+}^{(s)}, \ldots {d_{j_{k^+}}^+}^{(s)}}$ becomes

$$\begin{aligned}&e^{o(1)}d_{\max }^{2k^+k^- - 2k^\pm }\frac{\prod _{i \in K^+} {{d^+_{i}}}^{ {{d^+_{i}}}^{(s)}}\prod _{i \in K^-} {{d^-_{ i}}}^{ {{d^-_{ i}}}^{(s)}} }{m^{k^+k^- - k^\pm }m^{2(m-s)}} {{m-s} \atopwithdelims (){{d_{i_1}^-}^{(s)}, \ldots ,{d_{i_{k^-}}^-}^{(s)} } }\\&\quad {{m-s} \atopwithdelims (){{d_{j_1}^+}^{(s)}, \ldots ,{d_{j_{k^+}}^+}^{(s)} } }. \end{aligned}$$

$$\square $$

Combining Eq. (48) with Lemma 4.2, we are able to prove the desired result.

### Lemma 5.1

The probability that Algorithm 1 returns a failure is *o*(1).

### Proof

In the statement of Lemma 4.2, the fraction $\left( \frac{d_{\max }^{2}}{m}\right) ^{{k^+k^- - k^{\pm }}}$ is either 1 if $k^+k^- = k^{\pm }$ or smaller than $\frac{d_{\max }^2}{m}$ if $k^+k^- \ne k^\pm$. Since $k^\pm \le \min \left( k^-,k^+\right)$, $k^+k^- = k^\pm$ implies that $k^+=k^-=1$. Together $k^+=k^-=1$ and the conditions under which the algorithm can fail imply that $K^+ = K^-$. First we consider this case. Since $K^+=K^-=K^\pm =1$, we have ${d_{i_1}^-}^{(s)} = {d_{i_1}^+}^{(s)} = m-s$, and after plugging this into Eq. (49),

$$\begin{aligned} \mathbb {P}\left[ A_{{d_{i_1}^-}^{(s)}, {d_{i_1}^+}^{(s)}}\right] \le e^{o(1)} \frac{ {d_{i_1}^+}^{ m-s}{d_{i_1}^-}^{ m-s}}{m^{m-s} m^{m-s}} = o(1). \end{aligned}$$

Next, assume that $k^+k^- \ne k^\pm$, which implies that $\left( \frac{d_{\max }^{2}}{m}\right) ^{{k^+k^- - k^{\pm }}} \le \frac{d_{\max }^2}{m}$. We apply the multinomial theorem to obtain:

$$\begin{aligned} \sum _{k^- = 1}^{\max \left( m-s, d_{\max }\right) } \sum _{i_1, \ldots , i_{k^-} = 1}^{n} \prod _{i \in K^-} {{d^-_{ i}}}^{ {{d^-_{ i}}}^{(s)}}{{m-s} \atopwithdelims (){{d_{i_1}^-}^{(s)}, \ldots ,{d_{i_{k^-}}^-}^{(s)} } } = \left( d_1^- + \ldots + d_n^-\right) ^{m-s} \end{aligned}$$

and

$$\begin{aligned} \sum _{k^+ = 1}^{\max \left( m-s, d_{\max }\right) }\sum _{j_1,\ldots , j_{k^+} =1}^n \prod _{i \in K^+} {{d^+_{i}}}^{ {{d^+_{i}}}^{(s)}}{{m-s} \atopwithdelims (){{d_{j_1}^+}^{(s)}, \ldots ,{d_{j_{k^+}}^+}^{(s)} } } =\left( d_1^+ + \ldots + d_n^+\right) ^{m-s}. \end{aligned}$$

Plugging these into Eq. (48) yields

$$\begin{aligned}&\mathbb {P}\left[ \,\text {failure}\, \right] \le o(1) + e^{o(1)}\frac{d_{\max }^2}{m}\\&\quad \sum _{m-s=1}^{d_{\max }^2} \frac{\left( d_1^+ + \ldots d_n^+\right) ^{m-s}\left( d_1^- + \ldots d_n^-\right) ^{m-s}}{m^{m-s}m^{m-s}} \le o(1). \end{aligned}$$

$$\square $$

This proves the claim of Theorem 2.1 about the failure probability of Algorithm 1.

## Running Time of Algorithm 1

When implementing Algorithm 1, one has a certain freedom to decide how exactly to choose random samples with probability proportional to $P_{i,j}$. Our implementation of Algorithm 1 uses the three-phase procedure introduced for regular graphs in Ref. [39] and extended to non-regular undirected graphs in Ref. [3]. We also distinguish three phases depending on the algorithm step *r*; however, our sampling probability is proportional ${{d^+_{i}}}^{(r)}{{d^-_{ j}}}^{(r)}\left( 1-\frac{{d^+_{i}}{d^-_{ j}}}{2m}\right)$, and the corresponding criteria that determine the phase of the algorithm are different. We also rely on the principle that looking up an element in a list with a dictionary (a list with a hashing table) requires constant time and, therefore, one can check in constant time whether an edge (*i*, *j*) is already present in the graph constructed thus far. For more information about how constant-time lookup can be implemented in practice, we refer the reader to the classical text Ref. [30]. In what follows, we show that the expected running time of our algorithm is linear in the number of edges.

### Lemma 5.1

Algorithm 1 can be implemented in such a way that its expected running time is $\mathcal {O}\left( m\right)$ for graphical degree sequences $\textbf{d}$ with $d_{\max } = \mathcal {O}\left( m^{1/4-\tau }\right)$.

### Proof

*Phase 1.* Let $E$ be the list of edges constructed by the algorithm so far, and let *E* be supplied with an index dictionary. In the first phase, a random unmatched in- and out-stubs are selected. We may check whether this is an eligible pair in time $\mathcal {O}\left( 1\right) ,$ as this is the time needed to look up an entry in a dictionary. If eligible, the pair is accepted with probability proportional to $1-\frac{{d^+_{i}}{d^-_{ j}}}{2m}$ and (*i*, *j*) is added to $E$. We select edges according to this procedure until the number of unmatched in-stubs drops below $2d_{\max }^2$. This marks the end of phase 1. As a crude estimate, each eligible pair is accepted with probability at least $\frac{1}{2}$, and at most $\frac{1}{2}$ of all stub pairs are ineligible, see Lemma 3.2(*a*). Hence, creating one edge in phase 1 has an expected computational complexity of $\mathcal {O}\left( 1\right)$, and the total runtime of this phase is $\mathcal {O}\left( m\right)$.

*Phase 2.* In this phase, we select a pair of vertices instead of a pair of stubs. This requires us to keep track of the list of vertices with unmatched in-stubs/out-stubs. These lists are constructed in $\mathcal {O}\left( n\right)$ and can be updated in a constant time. Draw uniformly random vertices *i* and *j* from the lists of vertices with unmatched out-stubs and in-stubs correspondingly. Accept *i* (respectively *j*) with probability $\frac{{{d^+_{i}}}^{(r)}}{{{d^+_{i}}}^{(r)}}$ $\left( \frac{{{d^-_{ j}}}^{(r)}}{{{d^-_{ j}}}^{(r)}}\right)$. If both vertices are accepted, we check if (*i*, *j*) is an eligible edge in time $\mathcal {O}\left( 1\right)$. If the edge is eligible, it is accepted with probability $1-\frac{{d^+_{i}}{d^-_{ j}}}{2m}$. Phase 2 ends when the number of vertices with unmatched in-stubs or the number of vertices with unmatched out-stubs is less than $2d_{\max }$. Since every vertex with unmatched in-stubs (respectively out-stubs) has at most $d_{\max }$ unmatched in-stubs (out-stubs), this guarantees that the edge is eligible with probability at least $\frac{1}{2}$. To get a pair of accepted vertices, we need an expected number of $\mathcal {O}\left( d_{\max }^2\right)$ redraws. Thus, the construction of one edge is expected to take $\mathcal {O}\left( d_{\max }^2\right)$. As there are only $2d_{\max }^2$ unmatched in-stubs at the start of phase 2, atmost $d_{\max }^2$ edges are created in this phase. Thus, the expected running time of Phase 2 is $\mathcal {O}\left( d_{\max }^4\right)$.

*Phase 3.* At the beginning of this phase, a list ${\widetilde{E}}$ of all remaining eligible edges is constructed. At the start of phase 3, there are only $2d_{\max }$ vertices left with unmatched in-stubs or with unmatched out-stubs. Thus, ${\widetilde{E}}$ contains no more than $4d_{\max }^2$ edges. For each possible edge, we search if it is already in the list *E* in time $\mathcal {O}\left( 1\right)$ to check whether the edge creates a double edge or self-loop. Thus, constructing ${\widetilde{E}}$ takes $\mathcal {O}\left( d_{\max }^2\right)$. The rest of Phase 3 consist of picking a random element of ${\widetilde{E}}$ and accepting it with probability $\frac{{{d^+_{i}}}^{(r)}{{d^-_{ j}}}^{(r)}}{{d^+_{i}}{d^-_{ j}}}\left( 1-\frac{{d^+_{i}}{d^-_{ j}}}{2m}\right)$. This leads to an expected number of $\mathcal {O}\left( d_{\max }^2\right)$ repetitions to accept one edge. If an edge is accepted, it is removed from ${\widetilde{E}}$ and the values of ${{d^+_{i}}}^{(r)}$ and ${{d^-_{ j}}}^{(r)}$ are updated. After selecting an element of ${\widetilde{E}}$, it must be checked if ${{d^+_{i}}}^{(r)} > 0$ and ${{d^-_{ j}}}^{(r)}>0$. If this is not the case, the edge is not added to *E* and removed from ${\widetilde{E}}$. This continues until ${\widetilde{E}}$ is empty or $|E| = m$. This has expected running time of order $\mathcal {O}\left( d_{\max }^4\right)$ as there are $\mathcal {O}\left( d_{\max }^2\right)$ edges that are expected to be discarded or accepted in $\mathcal {O}\left( d_{\max }^2\right)$. Thus, the total running time of the algorithm is $\mathcal {O}\left( m\right) + \mathcal {O}\left( n\right) + \mathcal {O}\left( d_{\max }^4\right) + \mathcal {O}\left( d_{\max }^4\right) .$ As $d_{\max } = \mathcal {O}\left( m^{1/4 - \tau }\right)$, the running time is $\mathcal {O}\left( m\right)$.

We must also compute $P_{ij}$ at each step. Let $P_{ij}^{(r)}$ denote the probabilities that the edge (*i*, *j*) is added to *E* at step *r*. Then

51 $$\begin{aligned} P_{ij}^{(r)} = \frac{{{d^+_{i}}}^{(r)}{{d^-_{ j}}}^{(r)}\left( 1-\frac{{d^+_{i}}{d^-_{ j}}}{2m}\right) }{(m-r)^2 - \Psi _r\left( \mathcal {N}\right) }. \end{aligned}$$

The numerator ${{d^+_{i}}}^{(r)}{{d^-_{ j}}}^{(r)}\left( 1-\frac{{d^+_{i}}{d^-_{ j}}}{2m}\right)$ can be computed in a constant time. To determine the denominator in (51), remark that:

$$\begin{aligned}&\left[ (m-r+1)^2 - \Psi _{r+1}\left( \mathcal {N}\right) \right] - \left[ (m-r)^2 - \Psi _{r}\left( \mathcal {N}\right) \right] \\&\quad = \sum _{(u,v) \in E_{r+1}} {{d^+_{u}}}^{(r+1)} {{d^-_{ v}}}^{(r+1)}\left( 1-\frac{{d^+_{u}}{d^-_{ v}}}{2m}\right) -\sum _{(u,v) \in E_{r}} {{{d^+_{u}}}^{(r)}} {{{d^-_{ v}}}^{(r)}}\left( 1-\frac{{d^+_{u}}{d^-_{ v}}}{2m}\right) \\&\qquad + \sum _{(i,v) \in G_{\mathcal {N}_r}} {{{d^-_{ v}}}^{(r)}}\left( 1-\frac{{d^+_{i}}{d^-_{ v}}}{2m}\right) + \sum _{(u,j) \in G_{\mathcal {N}_r}}{{{d^+_{u}}}^{(r)}} \left( 1-\frac{{d^+_{u}}{d^-_{ j}}}{2m}\right) \\&\qquad + {{d^-_{ i}}}^{(r)}\left( 1-\frac{{d^+_{i}}{d^-_{ j}}}{2m}\right) + {{d^+_{j}}}^{(r)}\left( 1-\frac{{d^+_{i}}{d^-_{ j}}}{2m}\right) . \end{aligned}$$

At each step *r*, each of the terms in the latter expression can be updated in $\mathcal {O}\left( d_{\max }\right)$ operations. This allows us to determine the value of $P_{ij}^{(r)}$ in time $\mathcal {O}\left( d_{\max }\right)$. As the construction of one edge also takes at least $\mathcal {O}\left( d_{\max }\right)$ in every phase, this does not change the overall complexity of the algorithm. The initial value is

$$\begin{aligned} \Psi _0\left( \mathcal {N}\right) = m^2 -\sum _{i=1}^n {d^-_{ i}}{d^+_{i}} - \frac{\sum _{i=1}^n{d^-_{ i}}^2 \sum _{i=1}^n {d^+_{i}}^2 - \sum _{i=1}^n{d^-_{ i}}^2{d^+_{i}}^2}{2m}, \end{aligned}$$

which can be computed in $\mathcal {O}\left( n\right)$. As $n \le m$, this does not change the order of the expected running time, and hence, this completes the proof. $\square$

This lemma completes the proof of Theorem 2.1.

## Data Availability

Data sharing not applicable to this article as no datasets were generated or analysed during the current study.
